# Regulation of CSF and Brain Tissue Sodium Levels by the Blood-CSF and Blood-Brain Barriers During Migraine

**DOI:** 10.3389/fncom.2020.00004

**Published:** 2020-02-04

**Authors:** Hamed Ghaffari, Samuel C. Grant, Linda R. Petzold, Michael G. Harrington

**Affiliations:** ^1^Department of Mechanical Engineering, University of California, Santa Barbara, Santa Barbara, CA, United States; ^2^Department of Chemical and Biomedical Engineering, FAMU-FSU College of Engineering, Tallahassee, FL, United States; ^3^Neuroscience, Huntington Medical Research Institutes, Pasadena, CA, United States

**Keywords:** migraine, sodium, mathematical model, Sobol's method, Na^+^, K^+^-ATPase

## Abstract

Cerebrospinal fluid (CSF) and brain tissue sodium levels increase during migraine. However, little is known regarding the underlying mechanisms of sodium homeostasis disturbance in the brain during the onset and propagation of migraine. Exploring the cause of sodium dysregulation in the brain is important, since correction of the altered sodium homeostasis could potentially treat migraine. Under the hypothesis that disturbances in sodium transport mechanisms at the blood-CSF barrier (BCSFB) and/or the blood-brain barrier (BBB) are the underlying cause of the elevated CSF and brain tissue sodium levels during migraines, we developed a mechanistic, differential equation model of a rat's brain to compare the significance of the BCSFB and the BBB in controlling CSF and brain tissue sodium levels. The model includes the ventricular system, subarachnoid space, brain tissue and blood. Sodium transport from blood to CSF across the BCSFB, and from blood to brain tissue across the BBB were modeled by influx permeability coefficients *P*_*BCSFB*_ and *P*_*BBB*_, respectively, while sodium movement from CSF into blood across the BCSFB, and from brain tissue to blood across the BBB were modeled by efflux permeability coefficients $P_{BCSFB}^{\prime }$ and $P_{BBB}^{\prime }$, respectively. We then performed a global sensitivity analysis to investigate the sensitivity of the ventricular CSF, subarachnoid CSF and brain tissue sodium concentrations to pathophysiological variations in *P*_*BCSFB*_, *P*_*BBB*_, $P_{BCSFB}^{\prime }$ and $P_{BBB}^{\prime }$. Our results show that the ventricular CSF sodium concentration is highly influenced by perturbations of *P*_*BCSFB*_, and to a much lesser extent by perturbations of $P_{BCSFB}^{\prime }$. Brain tissue and subarachnoid CSF sodium concentrations are more sensitive to pathophysiological variations of *P*_*BBB*_ and $P_{BBB}^{\prime }$ than variations of *P*_*BCSFB*_ and $P_{BCSFB}^{\prime }$ within 30 min of the onset of the perturbations. However, *P*_*BCSFB*_ is the most sensitive model parameter, followed by *P*_*BBB*_ and $P_{BBB}^{\prime }$, in controlling brain tissue and subarachnoid CSF sodium levels within 3 h of the perturbation onset.

## Introduction

Migraine is ranked among the top five causes of disability in the world (Steiner et al., 2018). Although the exact underlying causes of migraine are not known, common triggers of migraine include dehydration, stress, sleep disorders, hunger, etc. Understanding the pathophysiology of migraine is challenging because migraine triggering is different for everyone. Many of the triggers of migraine change the sodium balance in the brain. Animal and human studies (Harrington et al., 2006, 2011; Abad et al., 2018a; Meyer et al., 2019) have revealed that migraine sufferers have higher levels of cerebrospinal fluid (CSF) and brain interstitial fluid (ISF) sodium than control groups, while there is no significant difference between blood concentration of sodium in migraineurs and healthy controls. Studies have indicated that elevated levels of ISF sodium increase neuronal excitability (Hodgkin and Katz, 1949; Arakaki et al., 2011), which subsequently results in migraine. Brain sodium levels ultimately derive from peripheral circulation. Sodium is exchanged between the blood and brain across two major blood-brain interfaces, namely the blood-brain barrier (BBB) and the blood-CSF barrier (BCSFB). The BBB is formed by specialized endothelial cells lining the cerebral microvasculature and controls sodium exchange between the ISF and blood, while the BCSFB is formed by choroid plexus epithelial cells and regulates sodium transport between ventricular CSF and blood. Transfer of sodium across the BBB and the BCSFB predominantly take places via active, hence transcellular mechanisms. However, sodium may be able to cross the BCSFB and the BBB via a paracellular route through tight junctions between epithelial cells at the BCSFB and between endothelial cells at the BBB (Hladky and Barrand, 2016).

It is believed that the BCSFB and BBB are highly responsible for maintaining ion homeostasis in the brain. Thus, a disturbance in sodium transport mechanisms at the BCSFB and/or BBB can alter CSF and brain tissue sodium concentrations. However, the relative contributions of the two interfaces in the regulation of brain sodium homeostasis have yet to be determined. In this work, we use mechanistic modeling to study the significance of the BCSFB and BBB in controlling brain tissue and CSF sodium levels. We develop a mathematical model consisting of four compartments: the ventricular system, subarachnoid space, brain tissue and blood. Net movement of sodium across the BCSFB and BBB through different active and passive transport mechanisms is modeled by influx and efflux permeability coefficients of the interfaces to sodium. Influx permeability coefficients of the BCSFB and BBB to sodium refer to sodium movement from blood to CSF and brain tissue, respectively, whereas efflux permeability coefficients of the BCSFB and BBB to sodium represent sodium movement from CSF and brain tissue to blood, respectively. We study the dynamics of sodium distribution in the brain following a perturbation in the influx and efflux permeabilities of the BCSFB and BBB to sodium. We then perform a global sensitivity analysis (GSA) to assess the significance of the BCSFB and BBB in controlling sodium concentrations in the brain tissue, ventricular CSF and subarachnoid CSF. Our results reveal that the influx permeability coefficient of the BCSFB to sodium is the most sensitive model parameter in controlling ventricular CSF sodium concentration. Depending on the time elapsed from perturbations of the permeability coefficients, brain tissue and subarachnoid space CSF sodium levels can be significantly controlled by the BCSFB and/or BBB.

The computational model presented in this study can not only shed light on the dynamics of sodium exchange between CSF, brain tissue and blood, but can also provide insight for future experimental studies. In addition, this work can potentially offer a new strategy to normalize the elevated levels of brain sodium in migraine sufferers and potentially treat migraines.

## Methods

### Model Development

**Figure 1:**
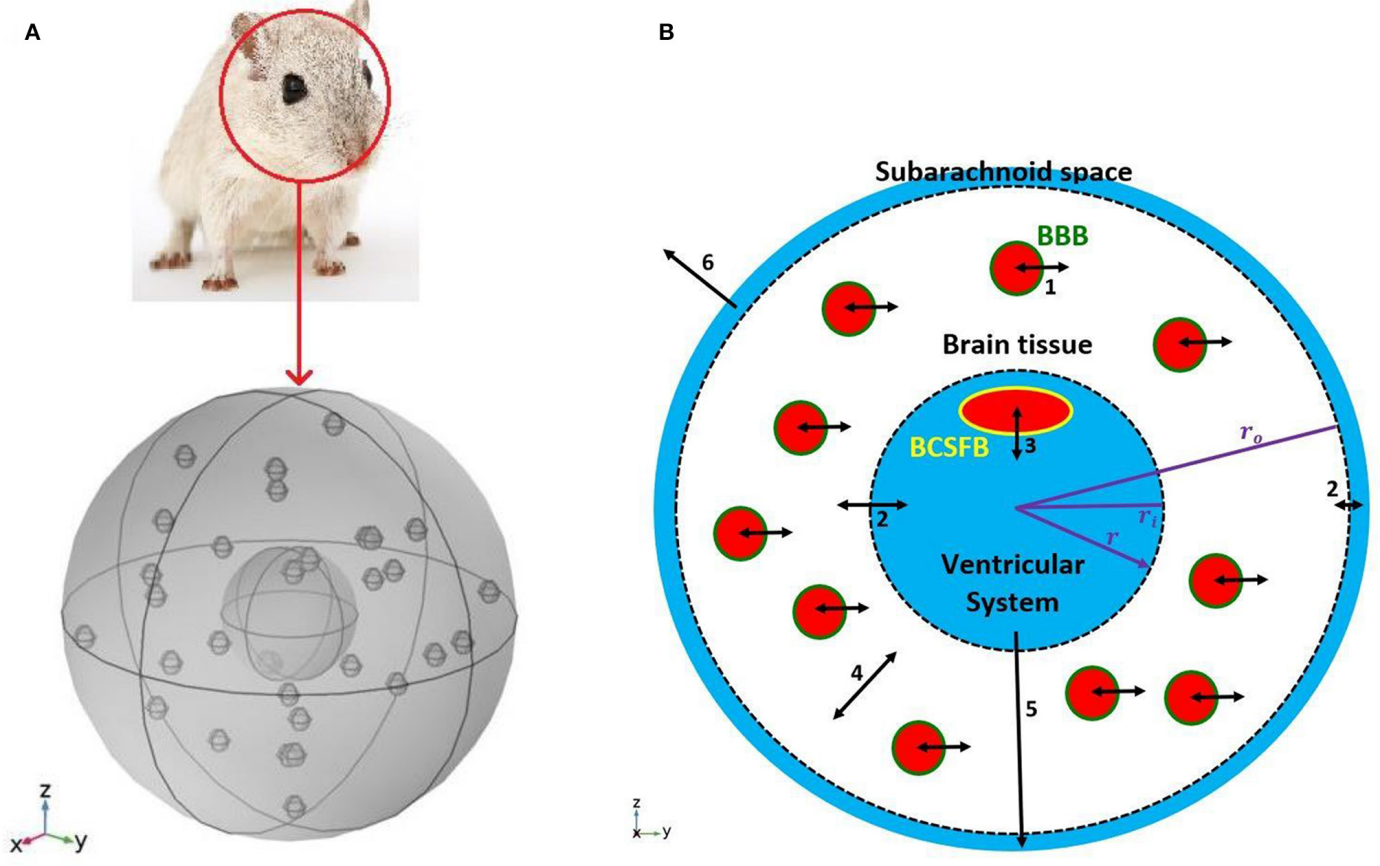
Schematic of the model. **(A)** A 3D model of a rat's brain. **(B)** A 2D view of the cross section of the 3D model. The inner circle, shown in blue, represents the ventricular system, while the outer ring, shown in blue, is subarachnoid space. The white region between two dashed circles is brain tissue. Blood vessels, shown in red filled circles, are distributed uniformly in the brain tissue. The green circular border which separates blood from the brain tissue is the BBB. The BCSFB which is depicted by a yellow ellipsoid separates blood from the ventricular CSF. Numbers in the figure specify the types and locations of sodium transport: 1. capillary-brain transport across the BBB; 2. exchange between CSF and ISF; 3. blood-CSF exchange across the BCSFB; 4. diffusive transport in the radial direction in the brain tissue; 5. transport by the CSF flow from the ventricular system to the subarachnoid space; 6 transport by the CSF flow from the subarachnoid space to the blood. Arrows 5 and 6 indicate CSF flow direction from the ventricular system to the subarachnoid space, and from the subarachnoid space to the blood. Although CSF flow has been modeled (Equations 1 and 2), the model does not include actual channels for transferring CSF flow between the ventricular system, subarachnoid space and blood. It should be noted that the size and number of the graphic symbols of blood vessels, as well as the size of the graphic symbol of choroid plexus (a.k.a BCSFB) do not represent their realistic values given in Table 1.

We modeled a rat's brain by three concentric spheres representing the ventricular system, brain tissue and subarachnoid space (Figure 1). Brain tissue was modeled as a single compartment. We assumed that blood vessels are distributed randomly, following a uniform distribution, throughout the brain tissue.

The inner sphere, which represents the ventricular system, includes the BCSFB. CSF is secreted by the BCSFB cells, a.k.a the choroid plexus epithelial cells, flows into the ventricular system, and then passes through small openings (foramina) into the subarachnoid space where it is absorbed through blood vessels into the bloodstream. It has also been suggested that a part of subarachnoid CSF moves into the brain along paravascular routes surrounding cerebral arteries, where it mixes with brain ISF and leaves the brain along veins (Iliff et al., 2012; Nedergaard, 2013). In the current model, we have ignored CSF flow from subarachnoid space to brain ISF (see section Discussion for further discussion of this subject). Thus, we have assumed that the CSF secretion rate is equal to the CSF absorption rate from the subarachnoid space to the blood. We have also assumed that sodium can be easily exchanged between the brain tissue and the CSF at the interface of brain tissue and the ventricular system, and at the contact surface of the subarachnoid space and brain tissue (dashed circles in Figure 1B). This is due to the negligible permeability of the contact surfaces. This transport can be considered as a diffusive transport with a very large diffusion coefficient, and is different from the convective CSF flow from the subarachnoid space to the ISF, which has been ignored in this work. Sodium is also exchanged between blood and brain tissue across the BBB, and can also diffuse in the brain tissue down its concentration gradient.

### Formulation of the Model

Ventricular and subarachnoid CSF sodium concentrations were modeled by ordinary differential equations (ODEs) represented by Equations (1) and (2), while the variation of sodium level across brain tissue was modeled by a partial differential equation (PDE), represented by Equation (3).

(1) $$\begin{array}{l} \frac{\partial C_{v}\left(t\right)}{\partial t}=\frac{{P_{BCSFB}A}_{BCSFB}}{V_{v}}C_{blood}-\frac{P_{BCSFB}^{\prime }A_{BCSFB}}{V_{v}}C_{v}\\ \;+\frac{P_{vb}A_{v}\lambda }{V_{v}}\left(\frac{C_{br}\left(t,r_{i}\right)}{f_{d}}-C_{v}\right)-\frac{Q_{csf}}{V_{v}}C_{v} \end{array}$$

(2) $$\begin{array}{l} \frac{\partial C_{s}\left(t\right)}{\partial t}=\frac{P_{sb}A_{s}\lambda }{V_{s}}\left(\frac{C_{br}\left(t,r_{o}\right)}{f_{d}}-C_{s}\right)+\frac{Q_{csf}}{V_{s}}C_{v}-\frac{Q_{csf}}{V_{s}}C_{s} \end{array}$$

(3) $$\begin{array}{l} \frac{\partial C_{br}\left(t,r\right)}{\partial t}={P_{BBB}A}_{BBB}C_{blood}-\frac{P_{BBB}^{\prime }A_{BBB}}{f_{d}}C_{br}\\ \;+\frac{\lambda }{{\rho f}_{d}r^{2}}\frac{\partial }{\partial r}\left(Dr^{2}\frac{\partial C_{br}}{\partial r}\right),\;r_{i}<r<r_{o} \end{array}$$

where *C*_*v*_, *C*_*s*_, *C*_*blood*_, *C*_*br*_ and *t* represent ventricular CSF sodium concentration, subarachnoid CSF sodium concentration, blood sodium concentration, sodium level in brain tissue and time, respectively. *C*_*v*_, *C*_*s*_, and *C*_*blood*_ are expressed in *mol ml*^−1^, while *C*_*br*_ is defined as moles of sodium per gram of brain (*mol g*^−1^). *C*_*br*_ includes sodium content in brain ISF and in brain cells. The ISF sodium concentration (*mol ml*^−1^) was estimated from the brain tissue sodium level (*mol g*^−1^) by (Smith and Rapoport, 1986)

(4) $$\begin{array}{l} C_{ISF}\left(t,r\right)=\frac{C_{br}\left(t,r\right)}{f_{d}} \end{array}$$

where *C*_*ISF*_ and *f*_*d*_ are the ISF sodium concentration and sodium distribution factor, respectively. The model's parameters are defined in Table 1.

**Table 1:** Physiological values of the model's parameters for an adult rat.

**Parameters**	**Description**	**Value**	**References**
*P*_*BCSFB*_	BCSFB influx permeability coefficient to sodium (from blood to CSF)	3.8 × 10^−5^ (*cm s*^−1^)	Smith and Rapoport, 1986
*A*_*BCSFB*_	Surface area of BCSFB	1 (*cm*^2^)	Smith and Rapoport, 1986
$P_{BCSFB}^{\prime }$	BCSFB efflux permeability coefficient to sodium (from CSF to blood)	6.9 × 10^−7^ *cm s*^−1^	Calculated
*V*_*s*_	Subarachnoid space volume	0.2 (*cm*^3^)	Smith and Rapoport, 1986; Brøchner et al., 2015
*V*_*v*_	Ventricular system volume	0.1 (*cm*^3^)	Smith and Rapoport, 1986; Brøchner et al., 2015
*V*_*b*_	Brain tissue volume	1.1 (*cm*^3^)	Sahin et al., 2011
*P*_*BBB*_	BBB influx permeability coefficient to sodium (from blood to brain tissue)	1.4 × 10^−7^ (*cm s*^−1^)	Smith and Rapoport, 1986
*A*_*BBB*_	Surface area of the BBB	140 (*cm*^2^ *g*^−1^)	Smith and Rapoport, 1986
$P_{BBB}^{\prime }$	BBB efflux permeability coefficient to sodium (from brain tissue to blood)	1.35 × 10^−7^ *cm s*^−1^	Calculated
*f*_*d*_	Sodium distribution factor	0.34 (*cm*^3^ *g*^−1^)	Smith and Rapoport, 1986
*D*	Diffusion coefficient of sodium in the brain ISF	1.15 × 10^−5^ (*cm*^2^ *s*^−1^)	Goodman et al., 2005
*Q*_*csf*_	CSF flow rate	3.6 × 10^−5^ (*cm*^3^ *s*^−1^)	Smith and Rapoport, 1986
*P*_*vb*_	Permeability coefficient of the contact surface of brain tissue and ventricular system to sodium	10^6^ (*cm s*^−1^)	A large value was used
*P*_*sb*_	Permeability coefficient of the contact surface of brain tissue and subarachnoid space to sodium	10^6^ (*cm s*^−1^)	A large value was used
λ	ISF/brain volume fraction	0.2 (dimensionless)	Smith and Rapoport, 1986; Lei et al., 2017
ρ	Rat brain density	1 (*g cm*^−3^)	Smith and Rapoport, 1986

The parameters *r*_*i*_ and *r*_*o*_, which specify the boundaries of brain tissue in Equation (3) and Figure 1B, were obtained via the relationships

(5) $$\begin{array}{l} V_{v}=\frac{4}{3}\pi {r_{i}}^{3} \end{array}$$

and

(6) $$\begin{array}{l} V_{v}+V_{b}=\frac{4}{3}\pi {r_{o}}^{3}, \end{array}$$

where *V*_*v*_ and *V*_*b*_ represent the ventricular system volume and brain tissue volume, respectively. *r*_*i*_ is the radius of the inner sphere representing the ventricular system, while *r*_*o*_ is the radius of the middle sphere that represents the outer boundary of the brain tissue (Figure 1B). The terms on the left-hand side of Equations (1) and (2) represent the rate of change of sodium concentration (*mol ml*^−1^) in the ventricular and subarachnoid CSF, respectively, while the term on the left-hand side of Equation (3) represents the rate of change of sodium level (*mol g*^−1^) in the brain tissue. The four terms on the right-hand side of Equation (1) represent sodium transport from the blood to the ventricular CSF, sodium movement from the ventricular CSF to the blood, exchange of sodium between the ventricular CSF and the brain tissue, and sodium loss from the ventricular system due to bulk flow of CSF from the ventricular system to the subarachnoid space, from left to right, respectively. The three terms on the right-hand side of Equation (2) denote exchange of sodium between the subarachnoid CSF and the brain tissue, sodium input to the subarachnoid CSF due to the bulk flow of CSF, and sodium loss from the subarachnoid space due to CSF absorption into the blood, from left to right, respectively. The three terms on the right-hand side of Equation (3) represent sodium transport from the blood to the brain tissue, sodium movement from the brain tissue to the blood, and diffusive transport of sodium across the brain tissue, from left to right, respectively.

The initial conditions for the ODEs (Equations 1 and 2) are given by (Kawano et al., 1992; Gomes et al., 2017)

(7) $$\begin{array}{l} C_{v}=C_{s}=145\;mM. \end{array}$$

We have also assumed that *C*_*blood*_ is 140 mM at steady state (Kawano et al., 1992).

Rates of exchange of sodium at the boundaries of Equation (3) are defined by

(8) $$\begin{array}{l} Q_{v}=P_{vb}A_{v}\lambda \left(C_{v}-\frac{C_{br}\left(t,r\right)}{f_{d}}\right)\;r=r_{i} \end{array}$$

(9) $$\begin{array}{l} Q_{s}=P_{sb}A_{s}\lambda \left(C_{s}-\frac{C_{br}\left(t,r\right)}{f_{d}}\right).\;r=r_{o} \end{array}$$

We used large values for *P*_*sb*_ and *P*_*vb*_ due to high permeability of the contact surfaces to sodium. Thus, the ISF sodium concentration is approximately in equilibrium with ventricular and subarachnoid sodium concentrations at the interface of brain tissue and CSF. It is important to note that passive transport of sodium across the boundaries of brain tissue and CSF is regulated by the concentration gradient between the CSF and brain ISF (Equations 8 and 9). Brain ISF sodium concentration is estimated from brain tissue sodium level by Equation (4). *A*_*v*_ and *A*_*s*_ in Equations (8) and (9) represent the contact surface area of the brain tissue and the ventricular system, and the contact surface area of the brain tissue and the subarachnoid space, respectively. The contact surfaces were modeled as concentric spheres with the radiuses of *r*_*i*_ and *r*_*o*_ (Figure 1). *A*_*v*_ and *A*_*s*_ were obtained by

(10) $$\begin{array}{l} A_{v}=4\pi {r_{i}}^{2} \end{array}$$

and

(11) $$\begin{array}{l} A_{s}=4\pi {r_{o}}^{2} \end{array}$$

where *r*_*i*_ and *r*_*o*_ were calculated from Equations (5) and (6) using the physiological values of *V*_*v*_ and *V*_*b*_ (Table 1). In this model, *A*_*v*_ and *A*_*s*_ were obtained to be 1 and 5.5 *cm*^2^, respectively, consistent with experimental estimates of the contact surfaces areas (Levinger, 1971; Dicke and Roth, 2016).

$P_{BCSFB}^{\prime }$ and $P_{BBB}^{\prime }$ were calculated assuming that the CSF sodium level is in equilibrium with the brain tissue sodium concentration at *t* = 0 (steady state):

(12) $$\begin{array}{l} C_{br}\left(t,r\right)=C_{v}\times f_{d}=C_{s}\times f_{d}.\;for\;r_{i}\leq r\leq r_{o} \end{array}$$

This assumption implies that there is no sodium exchange between the CSF and the brain tissue at the two contact surfaces of brain tissue and CSF at *t* = 0 (Olsen and Rudolph, 1955; Bito and Davson, 1966). The obtained values for $P_{BCSFB}^{\prime }$ and $P_{BBB}^{\prime }$ were 6.9 × 10^−7^ *cm s*^−1^ and 1.35 × 10^−7^ *cm s*^−1^, respectively. In order to assess the validity of the obtained value for $P_{BBB}^{\prime }$, we calculated the rate constant for total sodium efflux from the brain tissue to the blood, defined by $\frac{P_{BBB}^{\prime }A_{BBB}}{f_{d}}$ (Cserr et al., 1981). The average value of $\frac{P_{BBB}^{\prime }A_{BBB}}{f_{d}}$ was 5.5 × 10^−5^ *s*^−1^ in this work, which is consistent with the value of 1 × 10^−4^ *s*^−1^ reported by Cserr et al. (1981).

In section Results, we perform a local sensitivity analysis to investigate how perturbations in *P*_*BCSFB*_, *P*_*BBB*_, $P_{BCSFB}^{\prime }\;$or $P_{BBB}^{\prime }$ affect brain and CSF sodium concentrations. We also perform a global sensitivity analysis (GSA) to further analyze the significance of variations in the permeability coefficients in controlling the levels of sodium in the CSF and brain tissue. To solve the system of differential equations described by Equations (1)–(3), we discretize Equation (3) with respect to the variable *r* using the central difference approximation, and we approximate the time derivatives via backward differences. The main advantage of this fully implicit scheme, a.k.a. backward time central space, is that it is unconditionally stable.

### Global Sensitivity Analysis

Global sensitivity analysis (GSA) is a numerical method designed to analyze the impacts of uncertain parameters on a model's output. Compared to local sensitivity analysis, which assesses the changes of model response by making small perturbations to each parameter while keeping the remaining parameters unchanged, GSA analyzes the variations in the model output when all model parameters can vary simultaneously over specified ranges. In other words, GSA investigates how the uncertainty of the model's output is apportioned to variations in multiple model inputs. This feature makes GSA useful for understanding the contributions of uncertain model parameters to the variations of the model output. In this work, we use GSA to compare the importance of *P*_*BCSFB*_, *P*_*BBB*_, $P_{BCSFB}^{\prime }$, and $P_{BBB}^{\prime }$ in controlling brain tissue and CSF sodium concentrations, while taking into account the inter-subject variability in all of the model's parameters. We use a MATLAB toolbox for GSA, called SAFE (Pianosi et al., 2015). We perform Sobol's sensitivity analysis, which quantitively ranks the relative importance of the parameters by decomposing the model's output variance into the contributions associated with each model's input. Sobol's method, which has been widely applied to complex systems biology and pharmacology models (Kim et al., 2010; Sumner et al., 2012; Zhang et al., 2014, 2015; Arabghahestani and Karimian, 2017; Biliouris et al., 2018; Ghaffari and Petzold, 2018; Ghaffari et al., 2019b), calculates the first-order and total-effect sensitivity indices for each model parameter. The first-order indices (*S*_*i*_) measure the individual contributions of each input to the variance of the model output, while the total-effect indices (*S*_*Ti*_) represent the total contribution of the input, including its first-order effect and all higher-order interactions. The total-effect sensitivity indices can be used to identify unimportant model parameters. Non-influential parameters can be fixed at any value within their range of variability without significantly affecting the model response. In Sobol's sensitivity analysis technique, the model parameters that have total-effect sensitivity indices below 0.01 are often considered non-influential (Tang et al., 2006; Sin et al., 2011) (see Supplementary Information for further details).

## Results

**Figure 2:**
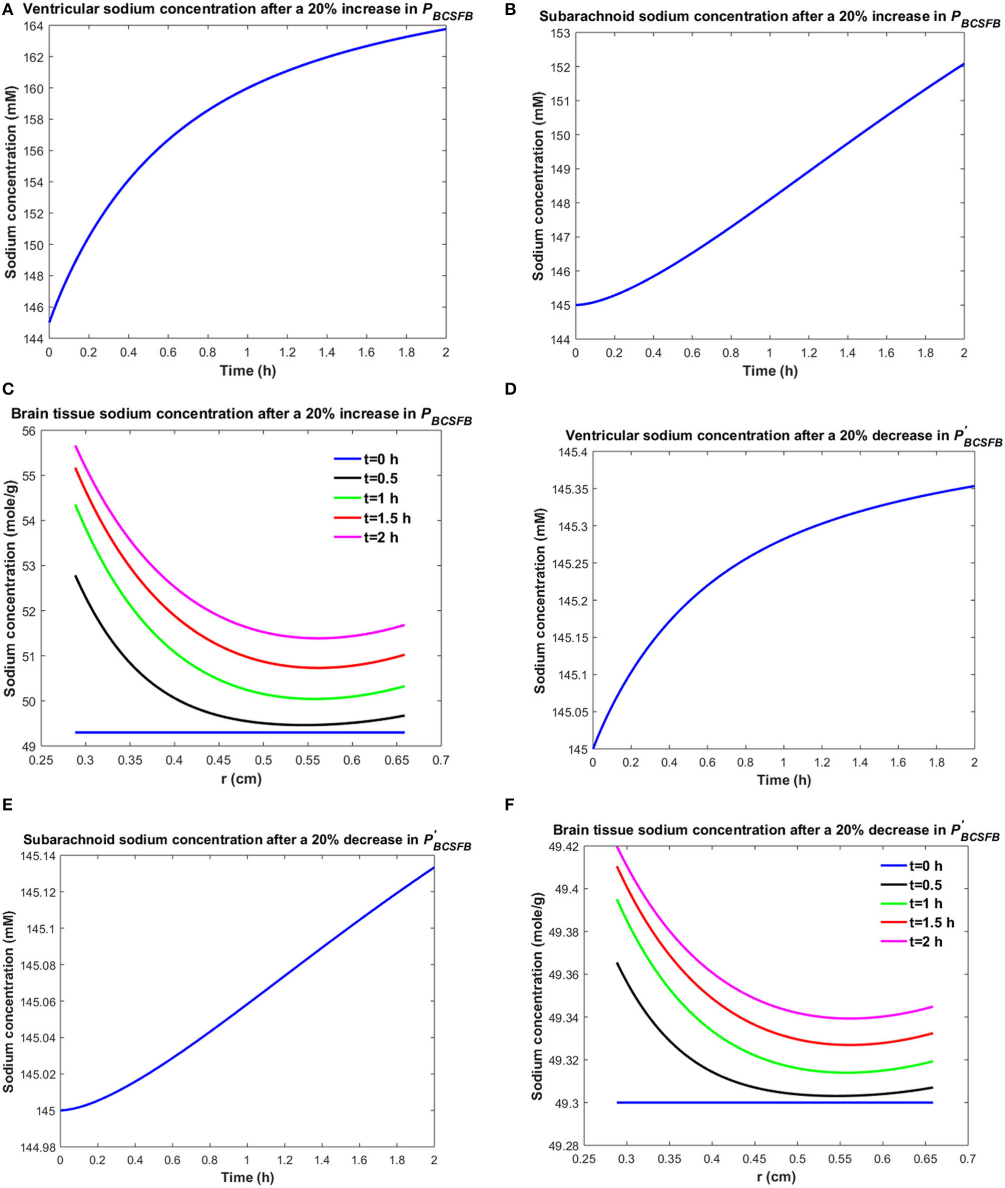
Variations of **(A)** *C*_*v*_ after increasing *P*_*BCSFB*_ by 20%, **(B)** *C*_*s*_ after increasing *P*_*BCSFB*_ by 20%, **(C)** *C*_*br*_ after increasing *P*_*BCSFB*_ by 20%, **(D)** *C*_*v*_ after decreasing $P_{BCSFB}^{\prime }$ by 20%, **(E)** *C*_*s*_ after decreasing $P_{BCSFB}^{\prime }$ by 20%, **(F)** *C*_*br*_ after decreasing $P_{BCSFB}^{\prime }$ by 20%.

It is believed that brain sodium homeostasis is highly regulated by the BCSFB and the BBB. Elevated levels of sodium in the CSF and brain tissue of migraine sufferers can be due to variations in the influx and/or efflux permeability coefficients of the BCSFB and/or the BBB to sodium. Heuristically, one may expect that the elevated CSF sodium concentration is due to increased transport of sodium from blood into CSF and/or decreased uptake of sodium from CSF into blood. Figure 2 shows the variations in brain tissue, ventricular and subarachnoid CSF sodium concentrations within 2 h after either a 20% increase in the influx permeability coefficient of the BCSFB to sodium (*P*_*BCSFB*_), or a 20% decrease in the efflux permeability coefficient of the BCSFB to sodium ($P_{BCSFB}^{\prime }$).

Ventricular CSF sodium concentration increases after a 20% rise in *P*_*BCSFB*_ or a 20% decrease in $P_{BCSFB}^{\prime }$ (Figures 2A,D). We assumed that sodium exchange between blood and CSF does not change blood concentration of sodium significantly, due to the large volume of blood compared to CSF. Thus, *C*_*blood*_ remains unchanged after changing the influx or efflux permeability coefficients of BCSFB to sodium. Figures 2C,F show that the elevated levels of sodium in the ventricular CSF lead to diffusion of sodium from CSF to brain tissue and distribution of sodium into the brain tissue over time (Smith and Rapoport, 1986; Murphy and Johanson, 1989). Sodium moves by bulk flow of CSF from the ventricular system to the subarachnoid space, where it can be exchanged between CSF and brain tissue. Subarachnoid CSF sodium concentration increases after increasing *P*_*BCSFB*_ or decreasing $P_{BCSFB}^{\prime }$ by 20% (Figures 2B,E). Our results indicate that ventricular CSF and subarachnoid CSF sodium concentration values at any given time point are more sensitive to variations of *P*_*BCSFB*_ than of $P_{BCSFB}^{\prime }$. Similarly, brain tissue sodium concentration values at any given time point and spatial location are more sensitive to changes of *P*_*BCSFB*_ than of $P_{BCSFB}^{\prime }$. These behaviors can be explained by the observation that steady state loss of ventricular CSF sodium is largely due to bulk flow of CSF from the ventricular system into the subarachnoid space rather than to sodium uptake by blood across the BCSFB (Equation 1 and physiological data in Table 1). However, the only source for sodium in the ventricular system is the choroid plexus epithelial cells, a.k.a BCSFB cells, at steady state. Thus, a 20% decrease in $P_{BCSFB}^{\prime }$ has a less significant impact than a 20% increase in *P*_*BCSFB*_ on CSF sodium content. It should be noted that we assume that there is no sodium exchange between the ventricular CSF and brain tissue at steady state (*t* = 0). Supplementary Videos 1, 2 show the variations of brain ISF, ventricular and subarachnoid sodium concentrations within 2 h after increasing *P*_*BCSFB*_ or decreasing $P_{BCSFB}^{\prime }\;$by 20%, respectively.

**Figure 3:**
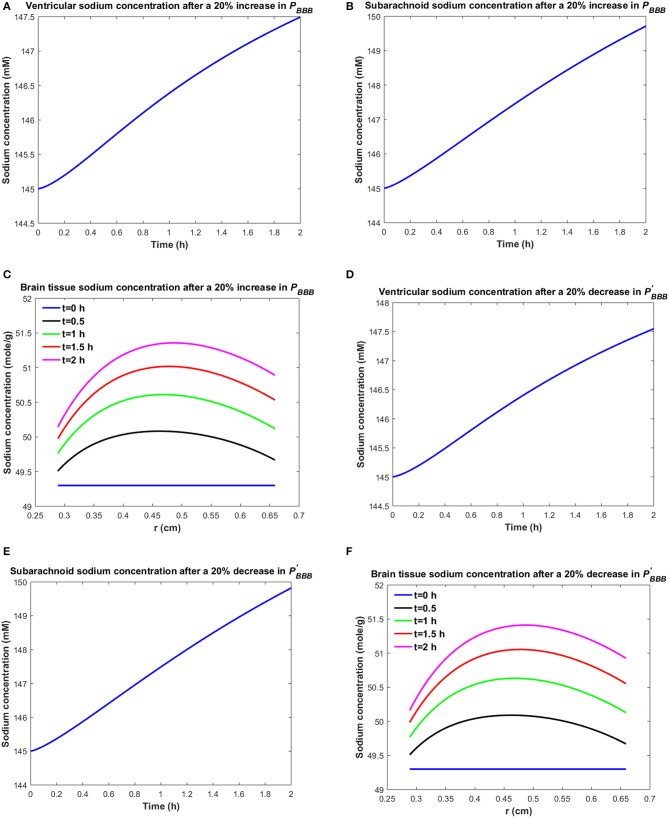
Variations of **(A)** *C*_*v*_ after increasing *P*_*BBB*_ by 20%, **(B)** *C*_*s*_ after increasing *P*_*BBB*_ by 20%, **(C)** *C*_*br*_ after increasing *P*_*BBB*_ by 20%, **(D)** *C*_*v*_ after decreasing $P_{BBB}^{\prime }$ by 20%, **(E)** *C*_*s*_ after decreasing $P_{BBB}^{\prime }$ by 20%, **(F)** *C*_*br*_ after decreasing $P_{BBB}^{\prime }$ by 20%.

Similarly, one may expect that the elevated brain tissue sodium levels during migraine are due to increased sodium transport from blood to brain tissue and/or reduced sodium uptake from brain tissue into blood. Figure 3 depicts the changes in ventricular CSF, subarachnoid CSF and brain tissue sodium levels within 2 h of either increasing the influx permeability coefficient of the BBB to sodium (*P*_*BBB*_) by 20%, or decreasing the efflux permeability coefficient of the BBB to sodium ($P_{BBB}^{\prime }$) by 20%.

A 20% increase in *P*_*BBB*_ or a 20% decrease in $P_{BBB}^{\prime }$ results in an accumulation of sodium in the brain tissue (Figures 3C,F). The elevated levels of sodium in the brain tissue increase sodium transport from brain tissue to the ventricular system and subarachnoid space (Figures 3A,B,D,E). Our results indicate that brain tissue, ventricular CSF and subarachnoid CSF sodium levels are almost equally sensitive to variations in *P*_*BBB*_ and $P_{BBB}^{\prime }$. Supplementary Videos 3, 4 show the changes in brain ISF, ventricular and subarachnoid sodium concentrations within 2 h after increasing *P*_*BBB*_ or decreasing $P_{BBB}^{\prime }\;$by 20%, respectively.

**Figure 4:**
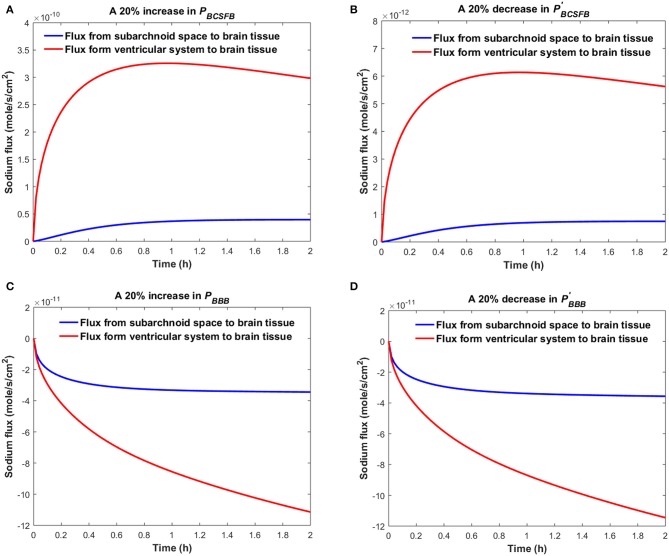
Comparison of sodium flux at the interface of the brain tissue and the ventricular system with sodium flux at the interface of the brain tissue and the subarachnoid space after **(A)** increasing *P*_*BCSFB*_, **(B)** decreasing $P_{BCSFB}^{\prime },$ **(C)** increasing *P*_*BBB*_, **(D)** decreasing $P_{BBB}^{\prime }$ by 20%. The positive sign of the flux indicates that sodium is diffusing from the CSF to the brain tissue, while the negative sign indicates that sodium is diffusing from the brain tissue to the CSF.

Figure 4 shows the sodium flux between the brain tissue and CSF at the interface of brain tissue and the ventricular system, and at the contact surface of brain tissue and the subarachnoid space, after perturbation of *P*_*BCSFB*_, *P*_*BBB*_, $P_{BCSFB}^{\prime }\;$, or $P_{BBB}^{\prime }\;$ by 20%. Our results indicate that sodium flux from the ventricular system to the brain tissue is larger than sodium flux from the subarachnoid space to the brain tissue.

Figures 2, 3 compare the variations in *C*_*v*_, *C*_*s*_, and *C*_*br*_ when a single parameter (i.e., *P*_*BCSFB*_, *P*_*BBB*_, $P_{BCSFB}^{\prime }$, or $P_{BBB}^{\prime }$) is perturbed and the rest of the parameters remain unchanged. However, in the case of migraines, all influx and efflux permeability coefficients can potentially vary. Additionally, Table 1 shows the average values of the physiological model's parameters. These values can change across a population of rats of the same type. Thus, we used GSA (Pianosi et al., 2015) to consider the effects of variations in all model parameters. In this regard, we assumed that physiological concentration of sodium in CSF and blood can vary within 5% of the *in vitro* values (i.e., *C*_*v*_ = *C*_*s*_ = 145 *mM, C*_*blood*_ = 140 *mM*), while the remaining independent model parameters (*P*_*BCSFB*_, *A*_*BCSFB*_, *V*_*s*_, *V*_*v*_, *V*_*b*_, *P*_*BBB*_, *A*_*BBB*_, *f*_*d*_, *D*, *Q*_*csf*_, λ, ρ) can vary within 25% of the *in vitro* values (Table 1). This is due to considering the impacts of intrinsic variations between a population of rats of the same type, and the effects of measurement errors in the estimations of physiological model parameters on our simulations. Following a uniform distribution, we sampled 10^5^ sets of parameters within their ranges of variability. We then calculated the dependent parameters, i.e., $P_{BCSFB}^{\prime }$ and $P_{BBB}^{\prime }$ for each set of parameters, assuming that the model is at steady state at *t* = 0. Each of these 10^5^ sets of parameters characterizes one healthy rat with different physiological parameters. We then assumed that *P*_*BCSFB*_, $P_{BBB},\;P_{BCSFB}^{\prime }$, and $P_{BBB}^{\prime }$ can undergo pathophysiological changes within 50% of their control values due to migraine triggers. We performed a GSA to investigate the significance of pathophysiological variations of *P*_*BCSFB*_, $P_{BBB},\;P_{BCSFB}^{\prime }$, and $P_{BBB}^{\prime }$ in influencing ventricular sodium concentration during episodic migraines. The model output was defined as the percent change of total ventricular sodium concentration within 2 h after perturbations of physiological *P*_*BCSFB*_, $P_{BBB},\;P_{BCSFB}^{\prime }$, and $P_{BBB}^{\prime }$:

$$\begin{array}{l} \text{Model Output}=\frac{\left(\frac{\int_{0}^{{\text{t}}_{\max}}{\text{C}}_{\text{v}}\text{ dt}}{{\text{t}}_{\max}}\right)\;-{\text{ C}}_{\text{v}}\left(\text{t}=0\right)}{{\text{C}}_{\text{v}}\left(\text{t}=\;0\right)} \end{array}$$

**Figure 5:**
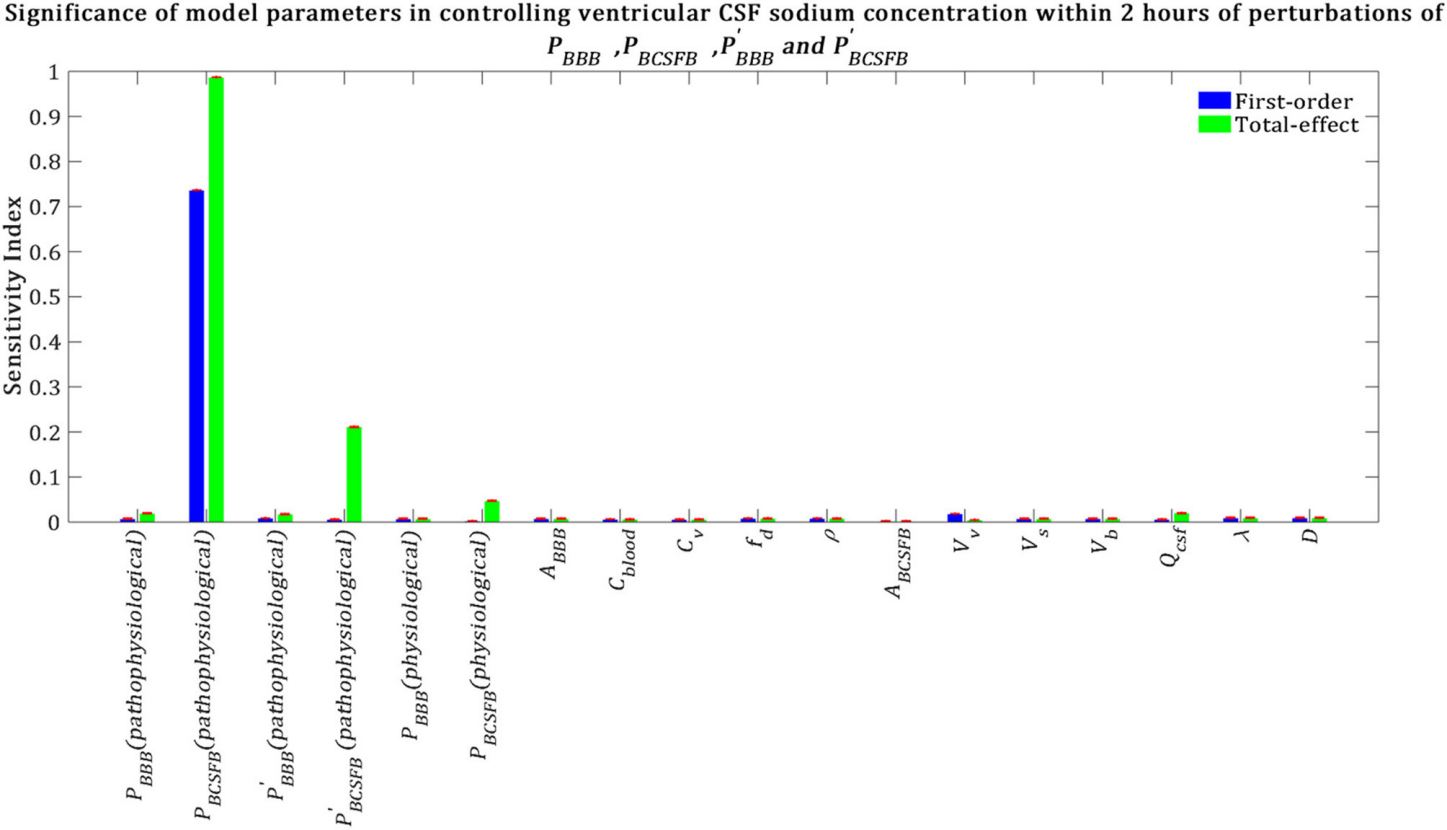
Sensitivity ranking of the model parameters. The model output was set to the time integral of *C*_*v*_ within 2 h after perturbation of the model's parameters. The blue bars represent first-order sensitivity indices, while the green bars show the total-effect sensitivity indices. The error bars, shown in red, indicate the bootstrap confidence intervals (95% confidence intervals) of the mean values.

Our results indicate that pathophysiological variation of *P*_*BCSFB*_ is much more important than that of $P_{BBB},\;P_{BCSFB}^{\prime }$, and $P_{BBB}^{\prime }$ in influencing ventricular CSF sodium concentration (Figure 5). It is important to note that each permeability coefficient is defined at two states: physiological and pathophysiological. A given permeability coefficient (e.g., *P*_*BCSFB*_) in the physiological and pathophysiological state is shown by *P*_*BCSFB*_ (*physiological*) and *P*_*BCSFB*_ (*pathophysiological*), respectively. Variations in *P*_*BCSFB*_(*physiological*) account for intrinsic variations between a population of rats of the same type and/or measurement errors in the estimations of the permeability coefficients. However, migraine triggers can cause a disturbance in sodium transport mechanisms at the BCSFB and/or BBB (Harrington et al., 2010; Gross et al., 2019). This implies that migraine triggers can change physiological permeability coefficients. *P*_*BCSFB*_(*pathophysiological*) represents the extent of variations in *P*_*BCSFB*_(*physiological*) due to migraine triggers. For a given rat with a given *P*_*BCSFB*_(*physiological*), different migraine triggers can change *P*_*BCSFB*_(*physiological*) differently; these changes are represented by *P*_*BCSFB*_(*pathophysiological*). Our results indicate that variations of *P*_*BCSFB*_(*physiological*) and *P*_*BBB*_(*physiological*) are much less important than those of *P*_*BCSFB*_(*pathophysiological*) and *P*_*BBB*_(*pathophysiological*) in influencing the percent change of total ventricular sodium concentration during migraines. This is mainly because the model output was defined as the percent change of total ventricular CSF sodium concentration between the pathophysiological and physiological states. These results suggest that the ventricular CSF sodium concentration is more sensitive to an alteration in homeostasis of the transporters which mediate sodium influx into CSF across the BCSFB than to a variation in homeostasis of the transporters which regulate sodium uptake from the CSF across the BCSFB. In addition, these results indicate that the BBB plays a much less important role than the BCSFB in regulation of the ventricular CSF sodium concentration. It is important to note that total-effect sensitivity indices, which account for total contribution of the inputs to variations in the model response, should be used to compare the significance of the model inputs in controlling the model output. *P*_*BCSFB*_ has a larger *S*_*Ti*_ than $P_{BBB},\;P_{BCSFB}^{\prime }$, and $P_{BBB}^{\prime }$, which indicates that *P*_*BCSFB*_ is a more influential parameter in the model.

**Figure 6:**
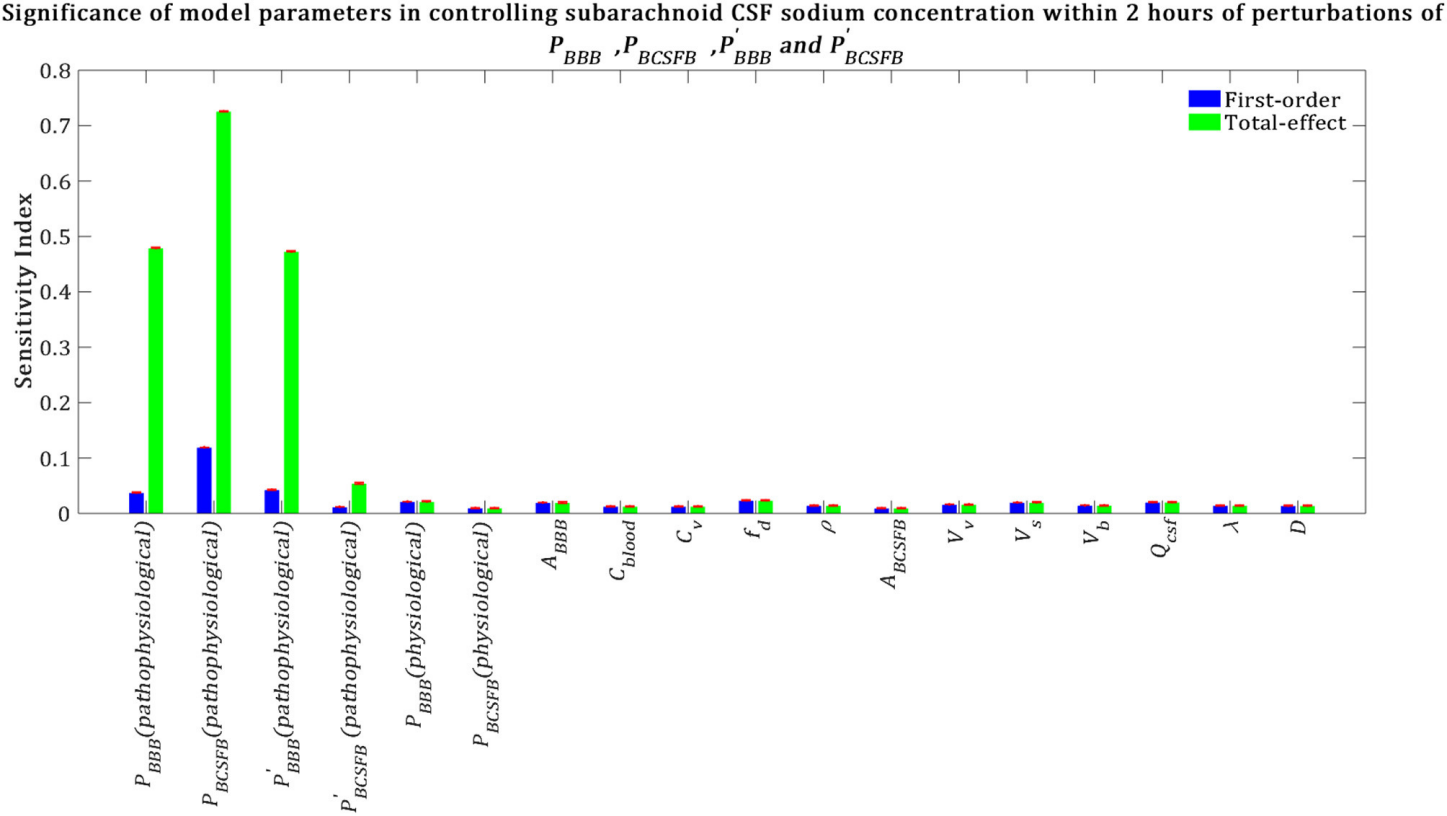
Relative significance of the model parameters in controlling subarachnoid CSF sodium concentration (*C*_*s*_) within 2 h of the perturbation onset (*t*_max_ = 2 *h*). The blue bars represent first-order sensitivity indices, while the green bars show the total-effect sensitivity indices. The error bars, shown in red, indicate the bootstrap confidence intervals (95% confidence intervals) of the mean values.

Total-effect sensitivity indices of some of the parameters are smaller than 0.01 (Figure 5). This means that the variations of these parameters do not influence the variance of the model output significantly; thus these parameters can be fixed at arbitrary values within their ranges (Tang et al., 2006; Sin et al., 2011). Figure 6 demonstrates the rank order of the model parameters when the model output was defined as the percent change of total subarachnoid sodium concentration within 2 h after perturbations of physiological *P*_*BCSFB*_, $P_{BBB},\;P_{BCSFB}^{\prime }$, and $P_{BBB}^{\prime }$ due to migraine triggers:

$$\begin{array}{l} \text{Model Output}=\frac{\left(\frac{\int_{0}^{{\text{t}}_{\max}}{\text{C}}_{\text{s}}\text{ dt}}{{\text{t}}_{\max}}\right)\;-{\text{ C}}_{\text{s}}\left(\text{t}=0\right)}{{\text{C}}_{\text{s}}\left(\text{t}=\;0\right)}. \end{array}$$

Our results indicate that subarachnoid CSF sodium concentration is highly sensitive to pathophysiological changes in *P*_*BCSFB*_, *P*_*BBB*_ and $P_{BBB}^{\prime }$ (Figure 6). The fact that pathophysiological variations of *P*_*BBB*_ and $P_{BBB}^{\prime }$ are more important in influencing subarachnoid sodium concentration than ventricular sodium concentration (Figures 5, 6) is because variations in *P*_*BBB*_ and $P_{BBB}^{\prime }$ not only can affect sodium exchange at the contact surface of subarachnoid CSF and brain tissue, but also can influence sodium exchange between the ventricular system and brain tissue, thus affecting the amount of sodium entering the subarachnoid space from the ventricular system.

We also performed a GSA to identify the influential parameters when the model output was the percent change in total level of brain sodium after 2 h of perturbations of the physiological *P*_*BCSFB*_, $P_{BBB},\;P_{BCSFB}^{\prime }$, and $P_{BBB}^{\prime }$ due to migraine triggers:

$$\begin{array}{l} \text{Model Output}\\ =\frac{\left(\int_{r_{i}}^{r_{o}}C_{br}\;4\pi r^{2}dr\right)-C_{br}\left(t=0\right)\times \left(total\;volume\;of\;brain\;tissue\;\right)}{C_{br}\left(t=0\right)\times \left(total\;volume\;of\;brain\;tissue\;\right)},\\ \;t\;=\;2\;h. \end{array}$$

**Figure 7:**
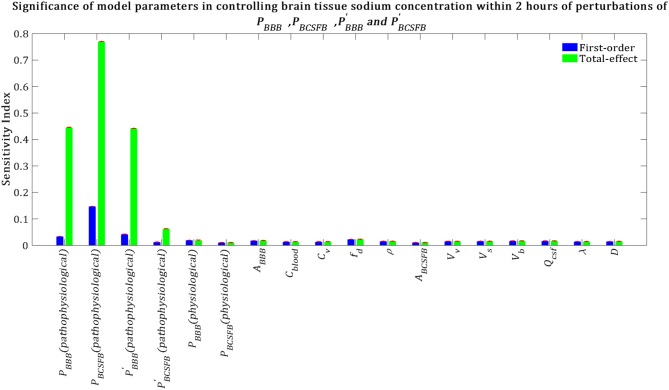
Relative importance of the model parameters in controlling brain tissue sodium levels within 2 h of the perturbation onset (*t*_max_ = 2 *h*). The blue bars represent first-order sensitivity indices, while the green bars show the total-effect sensitivity indices. The error bars, shown in red, indicate the bootstrap confidence intervals (95% confidence intervals) of the mean values.

Our results demonstrate that brain tissue sodium level is highly sensitive to pathophysiological variations in *P*_*BCSFB*_, *P*_*BBB*_, $P_{BBB}^{\prime }$ and $P_{BCSFB}^{\prime }$ in order of decreasing sensitivity (Figure 7). This result implies that sodium exchange between CSF and brain tissue at the contact surface of the ventricular system and brain tissue, as well as at the contact surface of the subarachnoid space and brain tissue can significantly influence brain sodium levels during migraine.

The above results were obtained after perturbing *P*_*BCSFB*_, $P_{BBB},\;P_{BCSFB}^{\prime }$, and $P_{BBB}^{\prime }$ at *t* = 0 and keeping them unchanged during the experiment time, i.e., *t* = 2 h. In order to investigate the impact of total experiment time on our results, we repeated our numerical experiments using different total experiment times including *t* = 1 min, *t* = 5 min, *t* = 10 min, *t* = 30 min, *t* = 1 h, and *t* = 3 h. Our results show that brain tissue, ventricular CSF and subarachnoid CSF sodium levels are mainly sensitive to pathophysiological variations in *P*_*BCSFB*_, *P*_*BBB*_, $P_{BBB}^{\prime }$, and $P_{BCSFB}^{\prime }$ (see Figures S1–S18). The significance of pathophysiological changes of *P*_*BCSFB*_, *P*_*BBB*_, $P_{BBB}^{\prime }$, and $P_{BCSFB}^{\prime }$ in influencing the ventricular CSF, subarachnoid CSF and brain tissue sodium levels at different total experiment times is shown in Table 2.

**Table 2:** Total-effect sensitivity indices of the permeability coefficients at different total experiment times.

	**Model output: ventricular CSF sodium**							**Model output: brain tissue sodium**							**Model output: subarachnoid CSF sodium**						
**Parameter *t*_**max**_**	**1 min**	**5 min**	**10 min**	**30 min**	**1 h**	**2 h**	**3 h**	**1 min**	**5 min**	**10 min**	**30 min**	**1 h**	**2 h**	**3 h**	**1 min**	**5 min**	**10 min**	**30 min**	**1 h**	**2 h**	**3 h**
*P*_*BBB*_	0.01	0.01	0	0	0.04	0.02	0.03	0.78	0.77	0.73	0.58	0.51	0.45	0.45	0.75	0.73	0.69	0.58	0.54	0.48	0.47
*P*_*BCSFB*_	0.93	0.95	0.95	0.97	0.97	0.98	0.99	0	0.13	0.23	0.48	0.67	0.77	0.82	0.01	0.18	0.29	0.48	0.62	0.73	0.79
$P_{BBB}^{\prime }$	0	0.01	0	0	0.05	0.01	0.03	0.78	0.77	0.73	0.58	0.49	0.44	0.46	0.75	0.73	0.69	0.58	0.53	0.47	0.48
$P_{BCSFB}^{\prime }$	0.2	0.21	0.2	0.2	0.2	0.21	0.2	0	0.01	0	0.02	0.03	0.06	0.1	0.01	0.01	0.01	0.01	0.03	0.05	0.09

Our results demonstrate that the ventricular CSF sodium concentration is highly sensitive to pathophysiological variations in *P*_*BCSFB*_, independent of experiment duration time. However, brain tissue and subarachnoid CSF sodium levels are more sensitive to pathophysiological variations of *P*_*BBB*_ and $P_{BBB}^{\prime }$ than pathophysiological variations of *P*_*BCSFB*_ at short total experiment times (such as 1, 5, 10, and 30 min). Pathophysiological variations of *P*_*BCSFB*_ become more important than variations of *P*_*BBB*_ and $P_{BBB}^{\prime }$ in controlling brain tissue and subarachnoid CSF sodium concentrations at longer experiment times (such as 1, 2, and 3 h). This implies that the BCSFB becomes more important in controlling brain tissue sodium homeostasis as time passes. This change in the significance of BCSFB and BBB in the regulation of brain tissue and subarachnoid CSF sodium levels over time is mainly due to the model structure, physiological model parameters and the model output expression. For instance, increasing brain tissue volume by 2-fold (which is not realistic) makes the BBB permeability coefficients (*P*_*BBB*_ and $P_{BBB}^{\prime }$) the most sensitive parameters in controlling brain tissue and subarachnoid CSF sodium levels, independent of the duration of the experiment (data not shown). This trend is due also in part to the fact that the ventricular CSF, whose sodium content is largely regulated by the BCSFB, would have enough time to influence sodium levels of its downstream compartments, including the brain tissue and the subarachnoid space.

**Figure 8:**
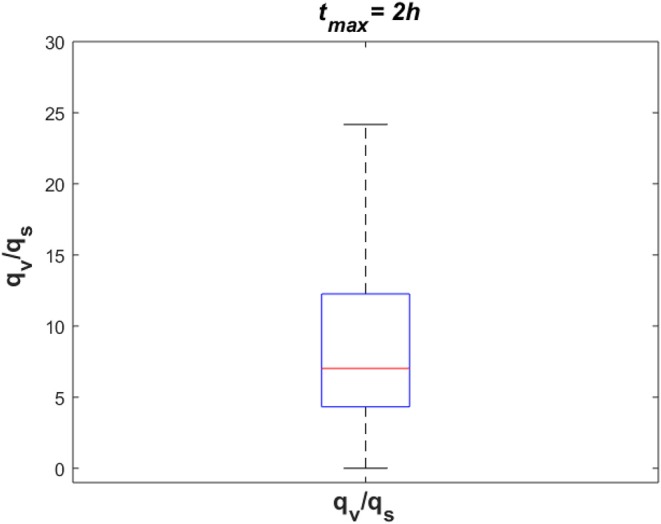
The ratio of absolute sodium flux at the interface of the ventricular system and the brain tissue (*q*_*v*_) to absolute sodium flux at the interface of the subarachnoid space and the brain tissue (*q*_*s*_). 10^5^ points were sampled randomly following a uniform distribution to generate this figure.

To investigate the dynamics of sodium exchange between the CSF and brain tissue at the interface of brain tissue and the ventricular system, and at the contact surface of brain tissue and subarachnoid space during an episode of migraine, we randomly sampled 10^5^ sets of parameters, following a uniform distribution over a 18-dimensional parameter space and compared the average absolute sodium flux (*q*_*v*_) between brain tissue and ventricular CSF, with the average absolute sodium flux (*q*_*s*_) between the brain tissue and subarachnoid CSF. The average absolute fluxes *q*_*v*_ and *q*_*s*_ are defined by

(13) $$\begin{array}{l} q_{v}=\frac{\int_{0}^{t_{\max}}\left|P_{vb}\lambda (C_{v}-\frac{C_{br}\left(t,r_{i}\right)}{V_{br}})\right|dt}{t_{\max}} \end{array}$$

(14) $$\begin{array}{l} q_{s}=\frac{\int_{0}^{t_{\max}}\left|P_{sb}\lambda (C_{s}-\frac{C_{br}\left(t,r_{o}\right)}{V_{br}})\right|dt\;}{t_{\max}}\;, \end{array}$$

where *t*_max_ = 2 *h*. Figure 8 shows the ratio of *q*_*v*_ to *q*_*s*_ for the 10^5^ randomly sampled parameters. Our results indicate that the ratio of *q*_*v*_ to *q*_*s*_ is >1 for the majority of the samples, which indicates that the absolute sodium flux at the interface of the ventricular system and the brain tissue is greater than the absolute sodium flux at the contact surface of the subarachnoid space and the brain tissue. Similar results were obtained for other total experiment times including *t*_max_ = 10 *min*, 30 *min*, 1 *h* (data not shown).

## Discussion

Previous studies (Harrington et al., 2006, 2011; Abad et al., 2018a; Meyer et al., 2019) have indicated that migraine sufferers have higher levels of CSF and brain tissue sodium than the control group. However, blood levels of sodium remain unchanged during migraine (Harrington et al., 2006). Under the hypothesis that these elevated sodium levels are due to variations in the influx and/or efflux permeability of the BCSFB and/or the BBB to sodium, we investigated the significance of variations in the influx and efflux permeabilities of the BCSFB and the BBB to sodium in influencing CSF and brain tissue sodium levels. In this regard, first we developed a computational model for sodium exchange between different brain compartments, i.e., blood, brain tissue, ventricular, and subarachnoid CSF. The model presented in this paper is similar in some respects to that of Smith and Rapoport (1986). However, there are two major differences between our model and theirs. First, our model includes the ventricular system and subarachnoid space as separate compartments. Thus, our model can distinguish between the ventricular and subarachnoid CSF, as well as provide insight into the dynamics of sodium exchange between the CSF and brain tissue at the interface of brain tissue and the ventricular system, and at the contact surface of brain tissue and the subarachnoid space. Second, we have proposed a more realistic model of brain tissue compared to previous studies (Davson and Welch, 1971; Collins and Dedrick, 1983; Smith and Rapoport, 1986). Unlike previous studies that modeled brain tissue as a rectangular sheet bathed on two opposite sides by CSF, we modeled brain tissue as the area between two concentric spheres. Concentric spheres are more similar to the real shape of a rat brain, which resembles an ellipsoid. As a result, the contact surface area of the brain tissue and the subarachnoid space is larger than that of the brain tissue and the ventricular system in our model. Thus, sodium exchange between the CSF and brain tissue at the two contact surfaces, as well as sodium diffusion in the brain tissue have been modeled more accurately in this work than in previous studies.

We performed a global sensitivity analysis to compare the significance of the BCSFB and the BBB in controlling CSF and brain sodium levels. Our results indicate that pathophysiological variations of the BCSFB influx permeability coefficient to sodium (*P*_*BCSFB*_) are more important than variations of the BCSFB efflux permeability coefficient ($P_{BCSFB}^{\prime }$), the BBB influx permeability coefficient (*P*_*BBB*_) and the BBB efflux permeability coefficient ($P_{BBB}^{\prime }$) to sodium in controlling ventricular CSF sodium concentrations. Brain tissue and subarachnoid CSF sodium levels are more sensitive to pathophysiological variations of *P*_*BBB*_ and $P_{BBB}^{\prime }$ than to variations of *P*_*BCSFB*_ when total experiment time is 1, 5, 10, and 30 min, while *P*_*BCSFB*_ becomes more important than *P*_*BBB*_ and $P_{BBB}^{\prime }$ in influencing brain tissue and subarachnoid CSF sodium levels when total experiment time is 1, 2, and 3 h. Overall, our results show that *P*_*BCSFB*_ plays an important role in the regulation of brain sodium homeostasis. *P*_*BCSFB*_ represents the net movement of sodium from blood to CSF, which is regulated by a variety of BCSFB sodium transporters, such as Na^+^, K^+^-ATPase (Davson and Segal, 1971; Wright, 1972; Huang et al., 2004; Leenen et al., 2015), ENaC (Van Huysse et al., 2012; Leenen et al., 2015), and NKCC1 (Steffensen et al., 2018). Thus, variations in *P*_*BCSFB*_ can be attributed to hyperactivity and/or hypoactivity of one or more of these sodium transporters. Our theoretical mechanism implies that the disturbed sodium homeostasis in the brain during a migraine is most likely due to overactivity of Na^+^, K^+^-ATPases at the BCSFB and the BBB (Harrington et al., 2010). Na^+^, K^+^-ATPase is a highly-conserved membrane protein which is expressed in all cells. One Na^+^, K^+^-ATPase mediates active transport of three sodium ions out of the cell for every two potassium ions entering the cell against the concentration gradients. We believe that disturbed homeostasis of Na^+^, K^+^-ATPase plays a key role in the pathophysiology of migraine (Gross et al., 2019), as many regulators of Na^+^, K^+^-ATPase, such as estrogen, adrenaline, insulin (Matsuda et al., 1993; Therien and Blostein, 2000), dopamine (Nishi et al., 1999; Hazelwood et al., 2008), glutamate (Nathanson et al., 1995), etc. are involved in the pathophysiology of migraine (see Harrington et al., 2010 for a comprehensive review). Furthermore, there are several lines of evidence supporting that CSF secretion as well as sodium transport from the BCSFB cells, a.k.a choroid plexus epithelial cells, to CSF is mostly mediated by Na^+^, K^+^-ATPases, which are expressed on the CSF-facing (apical) membrane of the BCSFB cells (Davson and Segal, 1970; Wright, 1972; Pollay et al., 1985). It has been shown that intracerebroventricular infusion of ouabain, an Na^+^, K^+^-ATPase inhibitor, at 10 *ug*/*day* decreases CSF sodium concentration by almost 8 mM in Wister rats on a high-salt diet (Huang et al., 2004). Ouabain can also reduce sodium transport from blood to CSF by 34 and 60% in frogs and rabbits, respectively (Davson and Segal, 1971; Wright, 1972). Thus, not only can the altered homeostasis of BCSFB Na^+^, K^+^-ATPases be a potential cause of the elevated CSF sodium concentration in a migraine, but also BCSFB Na^+^, K^+^-ATPase could be a candidate drug target to correct the elevated levels of sodium in CSF of migraine sufferers, potentially treating migraine. This hypothesis needs to be tested experimentally for different migraine triggers. ENaC is another sodium transporter which can play a key role in the regulation of CSF sodium levels. ENaC mediates passive sodium transport along a concentration gradient across the BCSFB. In Wister rats, ENaC is expressed at both membranes of BCSFB cells with a higher density at the CSF-facing (apical) membrane compared to the blood-facing (basolateral) membrane (Amin et al., 2009; Leenen, 2010). This suggests that ENaC may play a major role in sodium uptake from CSF into BCSFB cells (Wang et al., 2010). It should be noted that sodium movement through ENaC is likely to be unidirectional; thus, variations in the activity levels of ENaC at the apical membrane of BCSFB cells can potentially change $P_{BCSFB}^{\prime }$, while variations in ENaC activity levels at the basolateral membrane of BCSFB cells can possibly change *P*_*BCSFB*_. It is not known how the expression levels of ENaC on the different membranes of BCSFB cells are affected by migraine triggers. The other main BCSFB sodium transporter is NKCC1, which can regulate CSF production (Javaheri and Wagner, 1993) and sodium movement from blood to CSF (Steffensen et al., 2018). Overall, sodium transport from blood to CSF across the BCSFB is regulated by a variety of transporters, channels and proteins, whose interactions with each other are not well-understood. Further experimental studies are needed to elucidate the potential effects of various migraine triggers on the activity and expression levels of BCSFB Na^+^, K^+^-ATPase, ENaC, and NKCC1.

Our results suggest that the BBB can play a more important role than the BCSFB in the regulation of brain tissue and subarachnoid CSF sodium concentrations within 30 min of pathophysiological perturbations of *P*_*BCSFB*_, *P*_*BBB*_, $P_{BBB}^{\prime }$, and $P_{BCSFB}^{\prime }$. *P*_*BBB*_ and $P_{BBB}^{\prime }$ were used in the current model to simulate the net movement of sodium from blood to brain tissue, and from brain tissue to blood, respectively. Variations in *P*_*BBB*_ and $P_{BBB}^{\prime }$ can be attributed to altered homeostasis of the transporters, which mediate sodium movement across the BBB. The principal routes for sodium entry across the luminal membrane of the BBB endothelial cells are likely to be NKCC1 (Sun et al., 1995; O'donnell et al., 2004) and NHE 1,2 (Ennis et al., 1996), while sodium is mainly pumped out of the BBB endothelial cells into brain ISF by Na^+^, K^+^-ATPase (Betz et al., 1980; Del Pino et al., 1995; Hladky and Barrand, 2016). It has been suggested that sodium transport from the brain ISF into the BBB endothelial cells is mainly mediated by sodium-linked transporters of organic solutes, including those for amino acids (Hladky and Barrand, 2016). NHE 1,2 can also potentially contribute to sodium entry across the ISF-facing (abluminal) membrane of endothelial cells. However, the impacts of migraine triggers on the activity and expression levels of these sodium transporters are yet to be understood. Our results suggest that alterations of BBB sodium transporters homeostasis have more significant effects than variations of BCSFB sodium transporters homeostasis on brain tissue sodium levels within 30 min of the perturbation onset. It should be noted that our results were obtained using GSA, which gives us some insight into the importance of influx and efflux permeability of the BCSFB and the BBB to sodium in controlling CSF and brain tissue sodium by covering the entire parameter space, where all model parameters can vary within the specified ranges. Thus, in a rat model, the intrinsic variations between a population of rats of the same type were considered in this work.

This study has some limitations. First, for simplicity, we modeled the rat brain with three spheres. However, the real geometry of a rat brain is more complicated. A more realistic model of the brain and ventricles can provide a better understanding of the phenomenon under study. Second, we modeled the CSF with two well-mixed compartments, i.e., the ventricular system and the subarachnoid space. However, CSF flows through the lateral ventricles, the third ventricle, the cerebral aqueduct, the fourth ventricle, the cisterns and the subarachnoid space. Sodium concentration can vary slightly to significantly from one ventricle to another one and to the subarachnoid space. Thus, the current model can be improved to include all of the ventricles and subarachnoid space as separate compartments. CSF flow can be modeled using various numerical methods (Howden et al., 2008; Linninger et al., 2009; Arabghahestani et al., 2019; Kurtcuoglu et al., 2019). However, further information regarding the dynamics of sodium transport between different ventricles and adjacent brain tissues is needed. Furthermore, we assumed that there is no rate-limiting diffusion between the CSF and brain tissue at the two contact surfaces of the CSF and brain tissue. This results in instantaneous equilibrium between CSF sodium concentration and brain ISF sodium concentration at the contact surface of brain tissue and CSF (Smith and Rapoport, 1986). This assumption may not be true for some ependymal regions, such as those in the third ventricle as it has been shown that benzamil, an ENaC blocker can prevent sodium movement from the third ventricle CSF into brain tissue across the ependyma (Wang et al., 2010). Third, for simplicity we assumed that the value of the sodium distribution factor (*f*_*d*_) remains constant after perturbations of the BCSFB and the BBB permeability coefficients to sodium. Thus, we estimated the ISF sodium concentration by $\frac{C_{br}}{f_{d}}$ (Equation 4). This assumption implies that the ratio of extracellular sodium concentration to intracellular sodium concentration remains unchanged at any time after perturbations of the permeability coefficients. In other words, sodium is always distributed between the ISF and the brain cells in the ratio of their physiological sodium contents. Previous studies made a somewhat similar assumption to estimate the ISF sodium concentration from brain tissue sodium levels, using the cerebral distribution volume of sodium (Smith and Rapoport, 1986). The physiological value of *f*_*d*_ was found to be 0.34 ml/g using the average physiological ISF sodium concentration of 145 mM (Kawano et al., 1992) and the average brain tissue sodium concentration of 50 mM (= 50 × 10^−6^ mol/g) (Christensen et al., 1996). The obtained value of 0.34 ml/g for *f*_*d*_ in this work is the same as the value of the cerebral distribution volume of sodium (Smith and Rapoport, 1986). The dynamics of sodium exchange between the brain cells and the ISF can be better understood by adding the brain cells as a new compartment to the current model. Our model can be expanded to include brain cells once more information becomes available regarding the permeability coefficients of different types of brain cell to sodium. One approach to modeling of dynamic sodium exchange between the brain cells and the ISF is to use neuron models which are based on Hodgkin-Huxley type dynamics and extended to include time-dependent intracellular and extracellular sodium concentration (Dahlem et al., 2014; Hübel and Dahlem, 2014; Hübel et al., 2014). These dynamic models include differential equations for concentration of sodium, potassium and chloride. However, coupling these models with the current model may require modeling of further mechanisms that regulate potassium and chloride in the CSF and ISF. Fourth, we perturbed *P*_*BCSFB*_, $P_{BBB},\;P_{BCSFB}^{\prime }$, and $P_{BBB}^{\prime }$ at *t* = 0 and kept them unchanged during the experiment time. However, in reality the BCSFB and the BBB permeability coefficients likely change over time. Thus, the model presented in this study can be used to study the contribution of the BCSFB and the BBB to variations in the brain tissue and CSF sodium concentrations once there is more information about time-dependent variations of the BBB and BCSFB permeabilities to sodium during an episode of migraine with a particular trigger. Fifth, we assumed that diffusion is the major mechanism of sodium movement in the brain tissue. Although there are several lines of evidence supporting the existence of a convective transport mechanism called the glymphatic system in the brain (Iliff et al., 2012; Nedergaard, 2013), several aspects of glymphatic circulation, including whether interstitial transport is propagated by convective flow or diffusion (Jin et al., 2016; Holter et al., 2017), the identity of the ISF bulk flow driving forces (Asgari et al., 2016; Faghih and Sharp, 2018), and the role of astrocyte water permeability/aquaporin 4 (Jin et al., 2016) are still controversial. Furthermore, it is not well-understood how the proposed transport mechanisms are affected during migraine and how these mechanisms interact with the BBB to regulate ionic homeostasis in the brain. In this work, we ignored sodium transport between the CSF and brain ISF via convection, as it has been shown that diffusion (without convection) in the brain tissue is enough to account for many experimental transport studies in the brain parenchyma (Jin et al., 2016). Intuitively, we think that adding the convective CSF transport from the subarachnoid space to the brain ISF, based on the proposed glymphatic circulation, will increase the effects of subarachnoid CSF (in general CSF) on the brain tissue sodium levels, as the convective transport mechanism allows more sodium to be transported in a shorter amount of time compared to diffusive transport. Thus, the BCSFB would become more important in controlling brain tissue sodium levels. However, the exact extent of the contribution of the glymphatic system to the regulation of brain sodium homeostasis depends on not only the dynamical properties of the glymphatic system, such as the rate of glymphatic flow, the glymphatic efflux pathways and the ISF bulk flow driving force, but also the dynamic interactions between the glymphatic flow, the BBB and brain diffusive transport mechanisms. The current model can be expanded to include the convective CSF flow from the subarachnoid space to brain ISF once more information regarding the contribution of the glymphatic flow to the regulation of brain sodium homeostasis becomes available. Finally, we ignored water fluxes between the model compartments. Thus, the volumes of the model compartments remain unchanged during the experiment time. This is because variations of the permeability coefficients within the specified ranges in this study result in gradual changes in the brain ISF sodium concentration, which suggests that the ISF osmolality changes gradually. The gradual variations in the ISF osmolality give the brain cells enough time to adjust to the changes in the extracellular space; so that they can minimize the variations in their volume through regulating the influx and efflux of osmotically active solutes between the intracellular and extracellular fluids. Previous *in vitro* studies showed that the cultured cerebellar neurons and C6 rat glioma cells can exhibit isovolumetric regulation when the extracellular osmolality changes at a rate ≤1.8 and 3 mOsmol/kg/min, respectively (Mountian and Van Driessche, 1997; Tuz et al., 2001). The maximum possible rate of change of ISF sodium concentration in this work is 1.5 mM/min, equivalent to 1.5 mOsmol/kg/min. Thus, we believe that the brain cells, which make up 80% of total volume of the brain can significantly maintain their volume under the assumptions/conditions in our numerical simulation. This argument is in agreement with another experimental observation which suggests that a 50% decrease in the activity levels of Na^+^, K^+^-ATPase on the brain microvessels does not change total water content in the brain significantly (Moufarrij and Harik, 1989). Assuming that the brain tissue volume remains almost unchanged in this work, one can conclude that the CSF volume remains almost constant, due to the rigid confines of the skull. We have also assumed that the CSF secretion rate remains unchanged after pathophysiological variations of the influx and/or efflux permeability coefficients of the BCSFB to sodium. Although it has been suggested that there is a positive correlation between the CSF secretion and sodium transport rates across the BCSFB (Hladky and Barrand, 2016), it is not known how and to what extent water movement is linked to sodium transport in the BCSFB during migraine. Migraine is accompanied with a complex chain of biochemical changes in the CSF and brain which may contribute, together with sodium, to regulation of water movement across the BCSFB. For instance, it has been shown that CSF (and plasma) content of organic osmolytes, such as taurine and glutamate, which can significantly regulate brain cell volume homeostasis (Schousboe and Pasantes-Morales, 1992; Fisher et al., 2008), changes during migraine (Martinez et al., 1993; Cananzi et al., 1995; van Dongen et al., 2017; Abad et al., 2018b). However, it is yet to be determined how the variations in organic osmolyte levels can influence the osmotically driven water transport across the BCSFB. Thus, future experimental studies are needed to explore whether/how/to what extent the water movement rate depends on the sodium transport rate during migraine. The results presented in this work may vary depending on how the CSF flow rate changes during migraine. The current model can be extended to include dynamic water movement across the BCSFB once further information regarding the extent to which water movement is linked to sodium transport during migraine becomes available.

The fact that the CSF and brain tissue sodium levels are higher in migraine and in an analog of migraine in a rat model than the control groups (Harrington et al., 2006; Abad et al., 2018a) has relevance to the pain of migraine, since increasing extracellular sodium concentration immediately increases the firing rate of primary cultures of neurons (Arakaki et al., 2011). We propose that the increasing sodium concentration mainly arises from the BCSFB in the cerebral ventricles due to overactivity of Na^+^, K^+^-ATPases. When the higher CSF sodium concentration emerges from the fourth ventricle via the foramina of Luschka and Magendie, it meets the unmyelinated trigeminal nerves and the trigeminal ganglions. Unlike the cranial nerves, such as the facial nerve that are protected by their myelin, we predict that firing of the trigeminal nerve would increase in the presence of the elevated sodium concentration, with trigeminal pain as a consequence. Moreover, we also predict that this CSF efflux from the fourth ventricle may well be lateralized through one of the small foramina of Luschka, and hence would give rise to unilateral trigeminal stimulation. An alternative interpretation to the primary effect of CSF sodium in the initiation of migraine is that the sodium is a consequence of migraine. We consider this to be less likely, since we have recently demonstrated that specific inhibition of the BCSFB Na^+^, K^+^-ATPase protected the animal migraine model from nitroglycerin-triggered sensitization (Gross et al., 2019).

It is important to note that the altered Na^+^, K^+^-ATPase activity simultaneously shifts sodium and potassium. However, we have not modeled potassium since we originally found that sodium concentration changed in CSF, while potassium concentration did not change during migraines (Harrington et al., 2006). Furthermore, the potassium concentration in the ISF and CSF is maintained lower than in the remainder of the body by active astrocyte reuptake and, if potassium is not kept down, neurons will undergo apoptosis (Hertz and Chen, 2016). The mechanisms that regulate extracellular potassium concentration are substantially independent from Na^+^, K^+^-ATPase-driven changes in sodium. The current model can be improved to include more mechanisms once more experimental data for multiple ion and water fluxes and their regulation in conjunction with sodium becomes available.

## Conclusions

Our proposed mechanism for migraine suggests that a disturbance in brain sodium homeostasis causes migraine (Harrington et al., 2010). This sodium dysregulation is most likely due to variations in the influx and/or efflux permeability of the BCSFB and/or the BBB to sodium. The influx and efflux permeability of the BCSFB and the BBB to sodium represent the net effect of all transporters, channels and enzymes which contribute to movement of sodium across the interfaces. Thus, variations of the permeability coefficients can be caused by altered homeostasis of one or some of the sodium transport mechanisms at the interfaces. Unfortunately, understanding migraine pathophysiology is difficult, not only because the effects of various triggers on permeability of the BCSFB and the BBB to sodium are not known, but also because migraines have different triggers in different people. To approach this problem, we used mechanistic modeling together with global sensitivity analysis (GSA) to assess the relative importance of the BCSFB and the BBB in controlling CSF and brain tissue sodium levels. GSA provides insight into the significance of the BCSFB and the BBB in the regulation of brain sodium concentration when the exact extents of variations in the influx and efflux permeability coefficients of the BCSFB and the BBB to sodium are unknown. Our results show that the ventricular CSF sodium concentration is highly influenced by pathophysiological variations in the influx permeability coefficient of the BCSFB to sodium. Brain tissue and subarachnoid CSF sodium levels are more sensitive to pathophysiological variations in the BBB permeability coefficients than the BCSFB permeability coefficients to sodium at shorter total experiment times (such as 1, 5, 10, and 30 min), while the BCSFB becomes more important that the BBB in influencing total brain tissue and subarachnoid CSF sodium levels at longer experiment times (such as 1, 2, and 3 h). These results suggest that the efficacy of different migraine treatment strategies may depend on the time elapsed from migraine onset. This prediction needs to be tested experimentally for different models of migraines. This study prompts the hypothesis that increased influx permeability of the BCSFB to sodium caused by altered homeostasis of the enzymes which transport sodium from blood to CSF is the potential cause of elevated brain sodium levels in migraines. This hypothesis needs to be tested experimentally. The current model can be used to simulate sodium transport across the BBB, the BCSFB and the ependymal surfaces for a particular migraine trigger, given that the effects of the migraine trigger on the BBB and the BCSFB permeabilities are known. Further studies on the activity levels of different BCSFB and BBB sodium transporters during migraine episodes with different triggers can help better understand migraine pathophysiology.

## Data Availability Statement

All datasets generated for this study are included in the article/Supplementary Material.

## Author Contributions

HG conceived, designed, and performed the experiments. HG, SG, LP, and MH analyzed the results, wrote, and approved the paper.

### Conflict of Interest

The authors declare that the research was conducted in the absence of any commercial or financial relationships that could be construed as a potential conflict of interest.

## Supplementary Material

Supplementary Video 1 Sodium concentration in CSF and ISF after a 20% increase in BCSFB influx permeability coefficient.

Supplementary Video 2 Sodium concentration in CSF and ISF after a 20% decrease in BCSFB efflux permeability coefficient.

Supplementary Video 3 Sodium concentration in CSF and ISF after a 20% increase in BBB influx permeability coefficient.

Supplementary Video 4 Sodium concentration in CSF and ISF after a 20% decrease in BBB efflux permeability coefficient.

## Abbreviations

CSF: cerebrospinal fluid
BBB: blood-brain barrier
BCSFB: blood-CSF barrier
ISF: interstitial fluid
GSA: global sensitivity analysis.
