# Supplementary material for: Regulation of CSF and Brain Tissue Sodium Levels by the Blood-CSF and Blood-Brain Barriers During Migraine
Source: Front Comput Neurosci. 2020 Feb 4;14:4. doi: 10.3389/fncom.2020.00004 (PMC7010722; doi:10.3389/fncom.2020.00004)
Supplement: Supplementary file 1 [file Table_1.DOCX]

**Supplementary Information**

**Global Sensitivity Analysis**

In this work, we used a MATLAB toolbox called SAFE [1] to perform a global sensitivity analysis (GSA). SAFE implements several GSA methods such as the Elementary Effects Test, Regional Sensitivity Analysis, and Sobol's technique. Sobol’s method is a variance-based global sensitivity analysis technique which evaluates the sensitivity of the solutions with respect to the model parameters as well as the interactions between different parameters. Using the principles of variance decomposition, Sobol’s method ranks the parameters in terms of their importance. Given an integrable function $f$ over a *p*-dimensional parameter space $Ω^{p}$,

$y=f\left( x_{1}, x_{2},\ldots, x_{p} \right) (S1)$

Each parameter can vary within a finite range. Sobol’s method considers expansion of the response into a set of functions of increasing dimensionality,

$$f\left( x \right)=f_{0}+\sum_{i=1}^{p} f_{i}+\sum_{i=1}^{p} \sum_{j>i}^{p} f_{ij}+\ldots+f_{123\ldots p}, (S2)$$

where each individual term is a function of the parameters in its index. The total variance of the function output is defined by

$$D(y)=\int_{Ω^{p}} f^{2}\left( x \right) dx-\left( \int_{Ω^{p}} f\left( x \right) dx \right)^{2}. (S3)$$

Sobol’s technique is based on decomposition of the total variance $D$ into partial variances indicating the contributions from effects of individual parameters and combined effects of pairs of parameters. This decomposition is accomplished using the expansion of $f$ into terms of increasing dimensions (Eq. S2),

$$D\left( y \right)=\sum_{i=1}^{p} D_{i}\left( y \right)+\sum_{i=1}^{p} \sum_{j>i}^{p} D_{ij}\left( y \right)+\ldots+D_{123\ldots p}\left( y \right) . (S4)$$

According to Sobol’s method, the first-order sensitivity index for each parameter is given by

$$S_{i}=\frac{D_{i}\left( y \right)}{D\left( y \right)} , (S5)$$

The first-order sensitivity index accounts for the main individual contribution of each model parameter to the variance of the model output. The Sobol’s total-effect index, on the other hand, represents total contribution of the input to the response variation. The total-effect index for parameter $x_{i}$ is calculated by the sum of all sensitivity indices which have $i$ in their index

$$S_{Ti}=S_{i}+\sum_{i\neq j} S_{ij}+\sum_{i\neq j,i\neq l,j<l} S_{ijl}+\ldots(S6)$$

Based on Sobol’s approach, the necessary and sufficient condition for parameter $x_{i}$ to be a noninfluential factor is $S_{Ti}=0.$ However, previous studies have indicated that a parameter can be considered noninfluential if its total-effect sensitivity index is smaller than 0.01, and significantly smaller than total-effect sensitivity indices of the rest of the parameters [2-4].


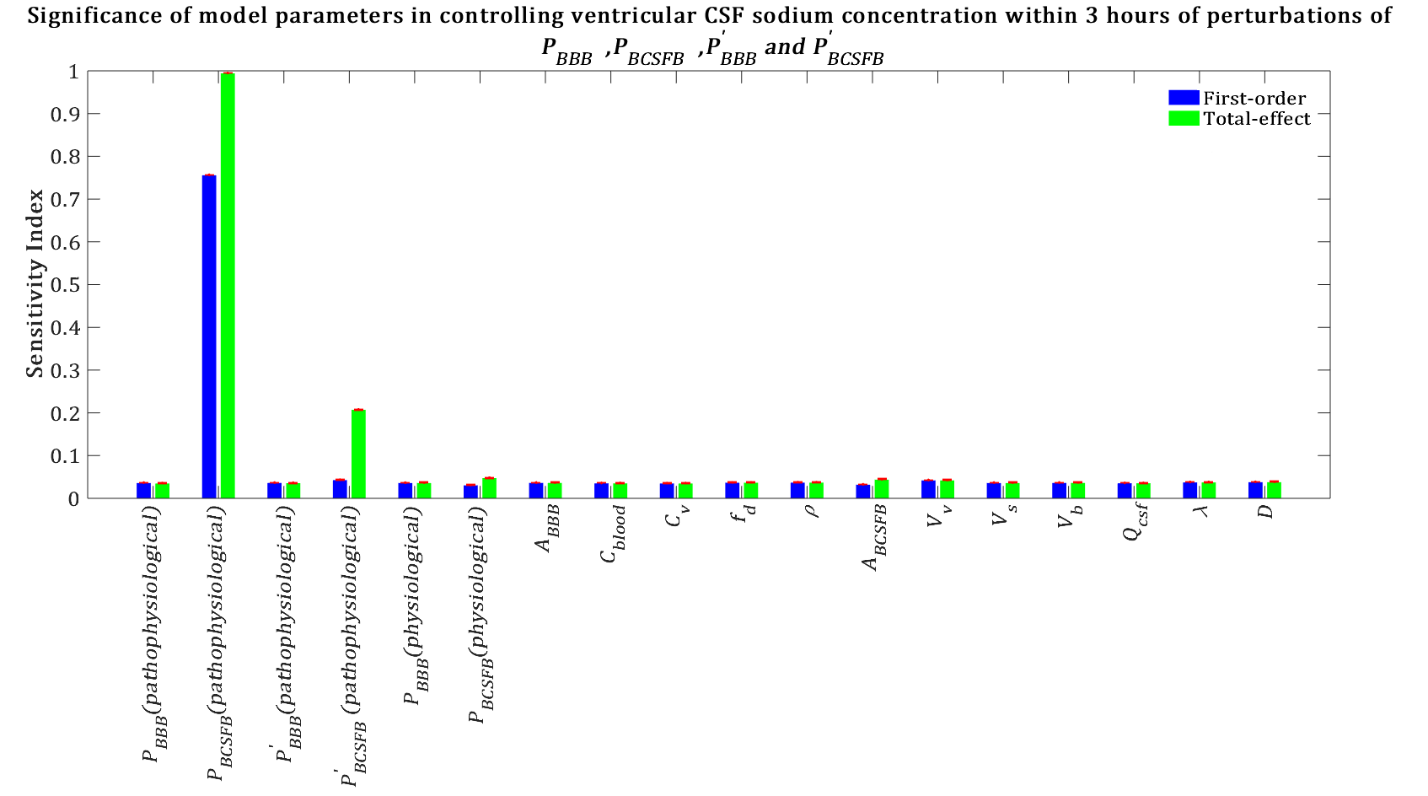


Figure S1. Relative significance of the model parameters in controlling ventricular CSF sodium concentration ($C_{v}$) within 3 hours of the perturbation onset ($t_{max}=3 h$). The blue bars represent first-order sensitivity indices, while the green bars show the total-effect sensitivity indices. The error bars, shown in red, indicate the bootstrap confidence intervals (95% confidence intervals) of the mean values.


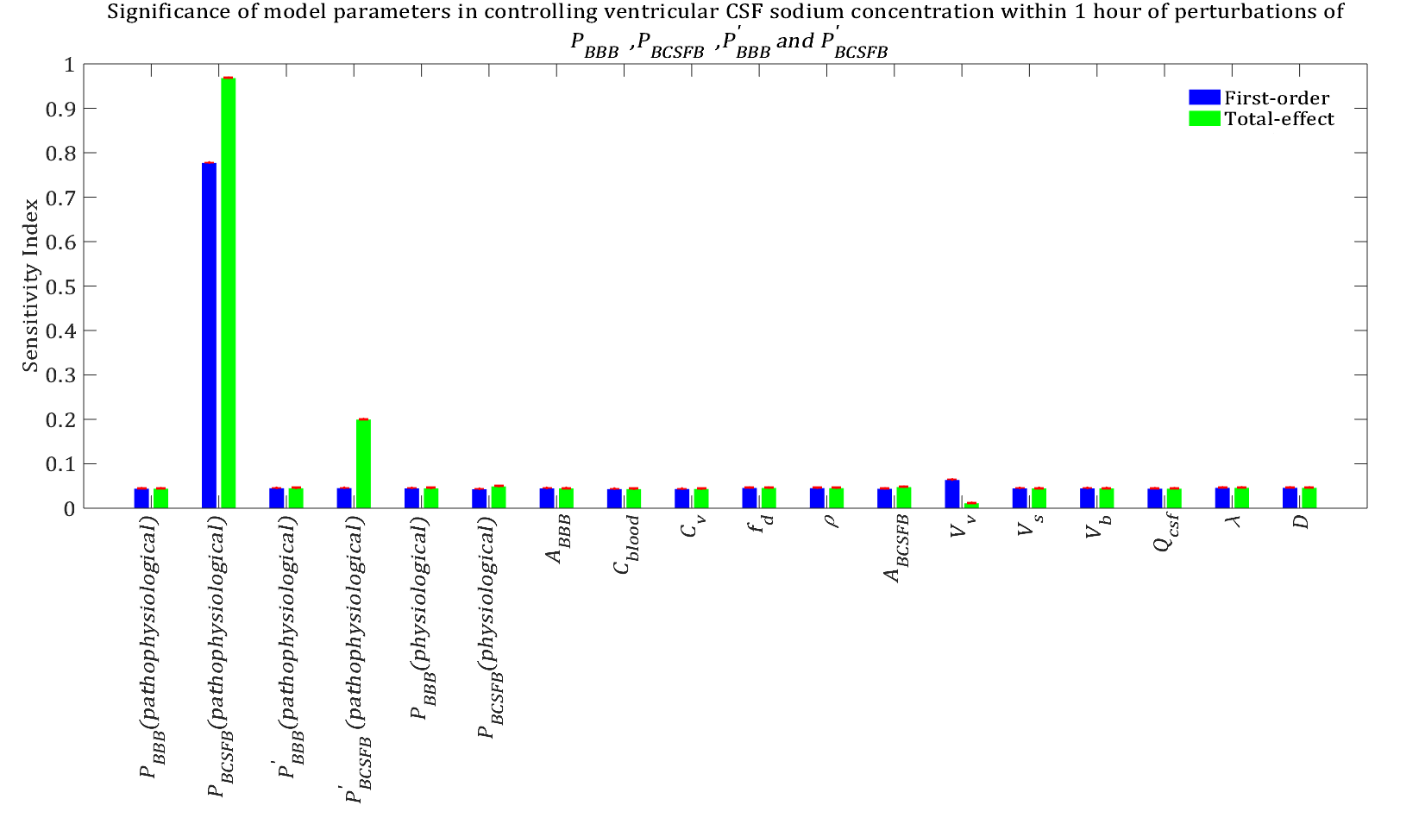


Figure S2. Relative significance of the model parameters in controlling ventricular CSF sodium concentration ($C_{v}$) within 1 hour of the perturbation onset ($t_{max}=1 h$). The blue bars represent first-order sensitivity indices, while the green bars show the total-effect sensitivity indices. The error bars, shown in red, indicate the bootstrap confidence intervals (95% confidence intervals) of the mean values.


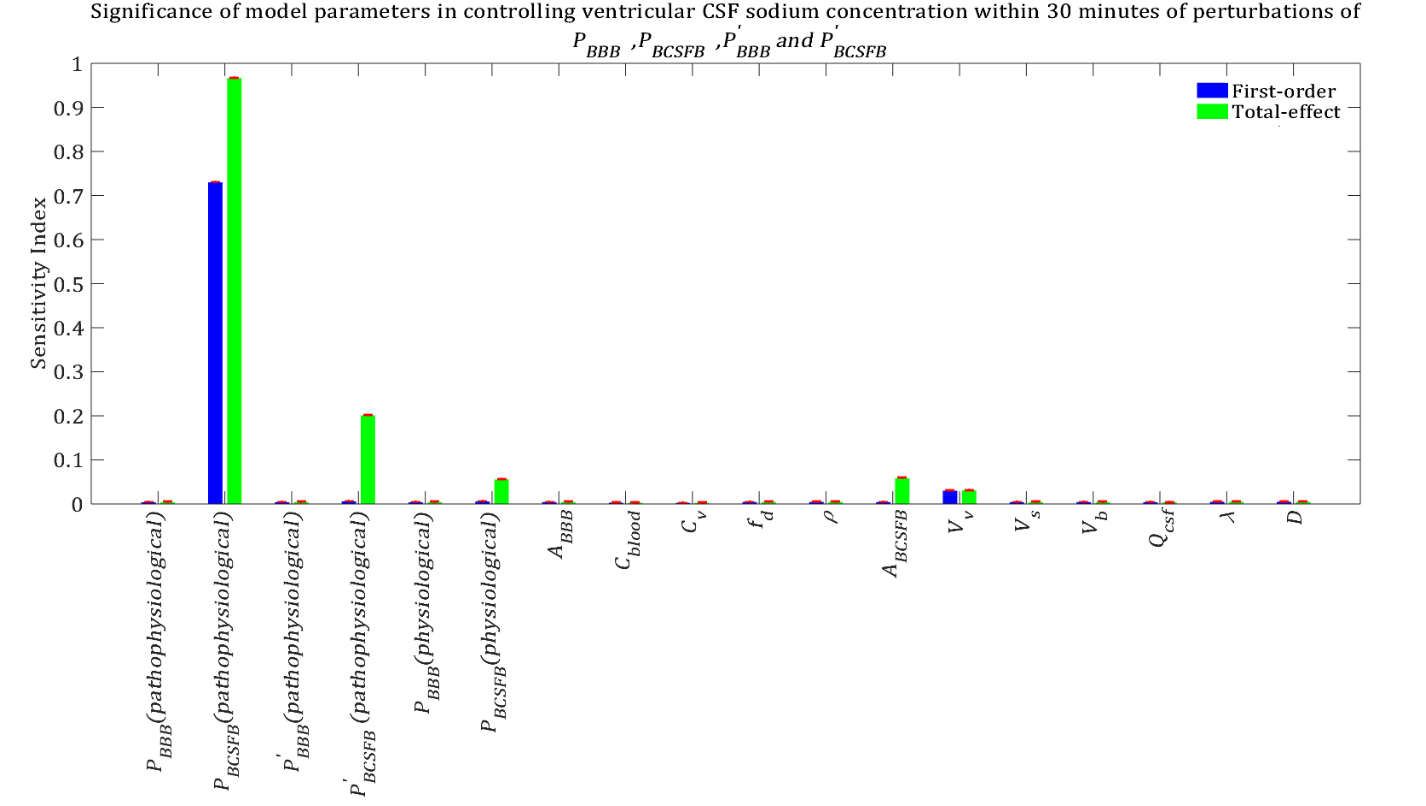


Figure S3. Relative significance of the model parameters in controlling ventricular CSF sodium concentration ($C_{v}$) within 30 minutes of the perturbation onset ($t_{max}=30 m$). The blue bars represent first-order sensitivity indices, while the green bars show the total-effect sensitivity indices. The error bars, shown in red, indicate the bootstrap confidence intervals (95% confidence intervals) of the mean values.


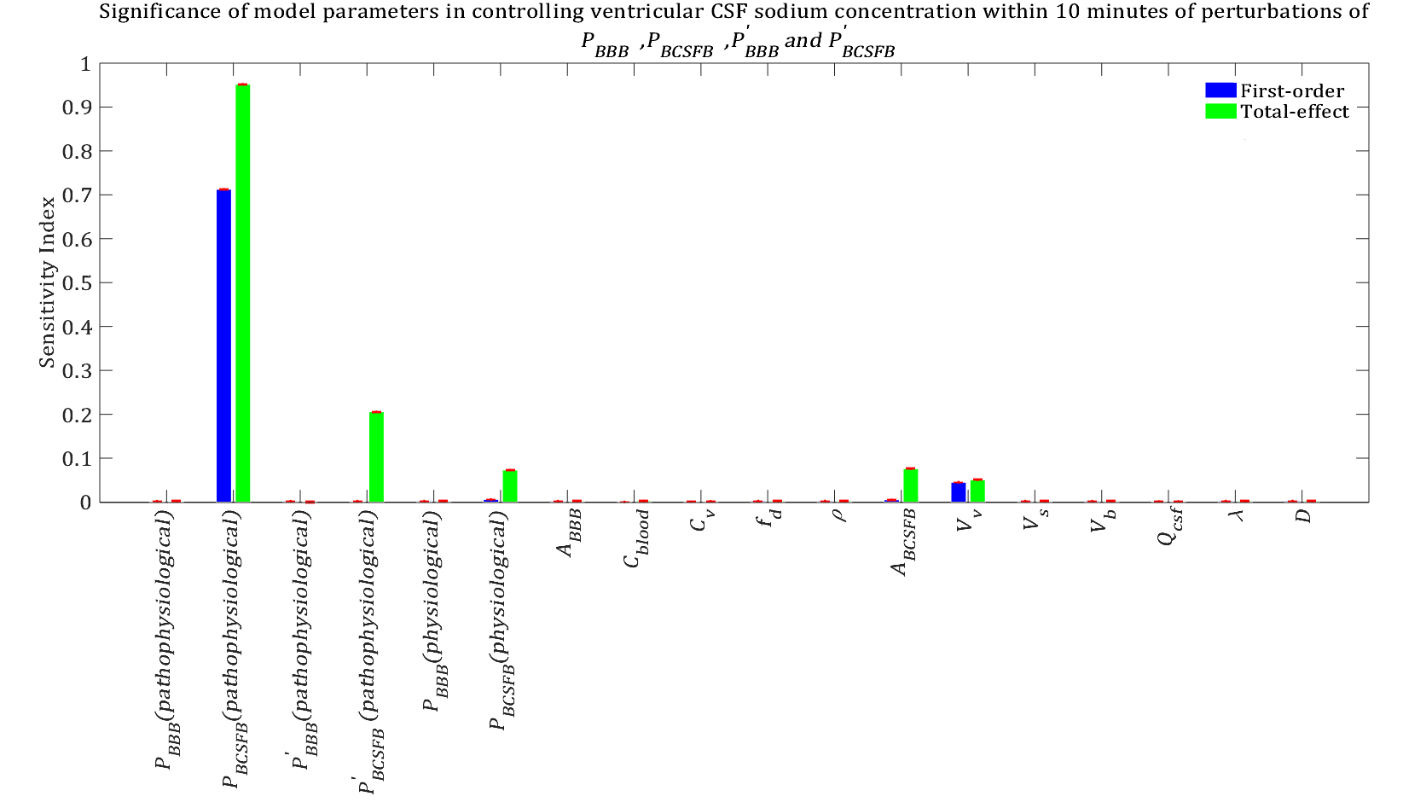


Figure S4. Relative significance of the model parameters in controlling ventricular CSF sodium concentration ($C_{v}$) within 10 minutes of the perturbation onset ($t_{max}=10 min$). The blue bars represent first-order sensitivity indices, while the green bars show the total-effect sensitivity indices. The error bars, shown in red, indicate the bootstrap confidence intervals (95% confidence intervals) of the mean values.


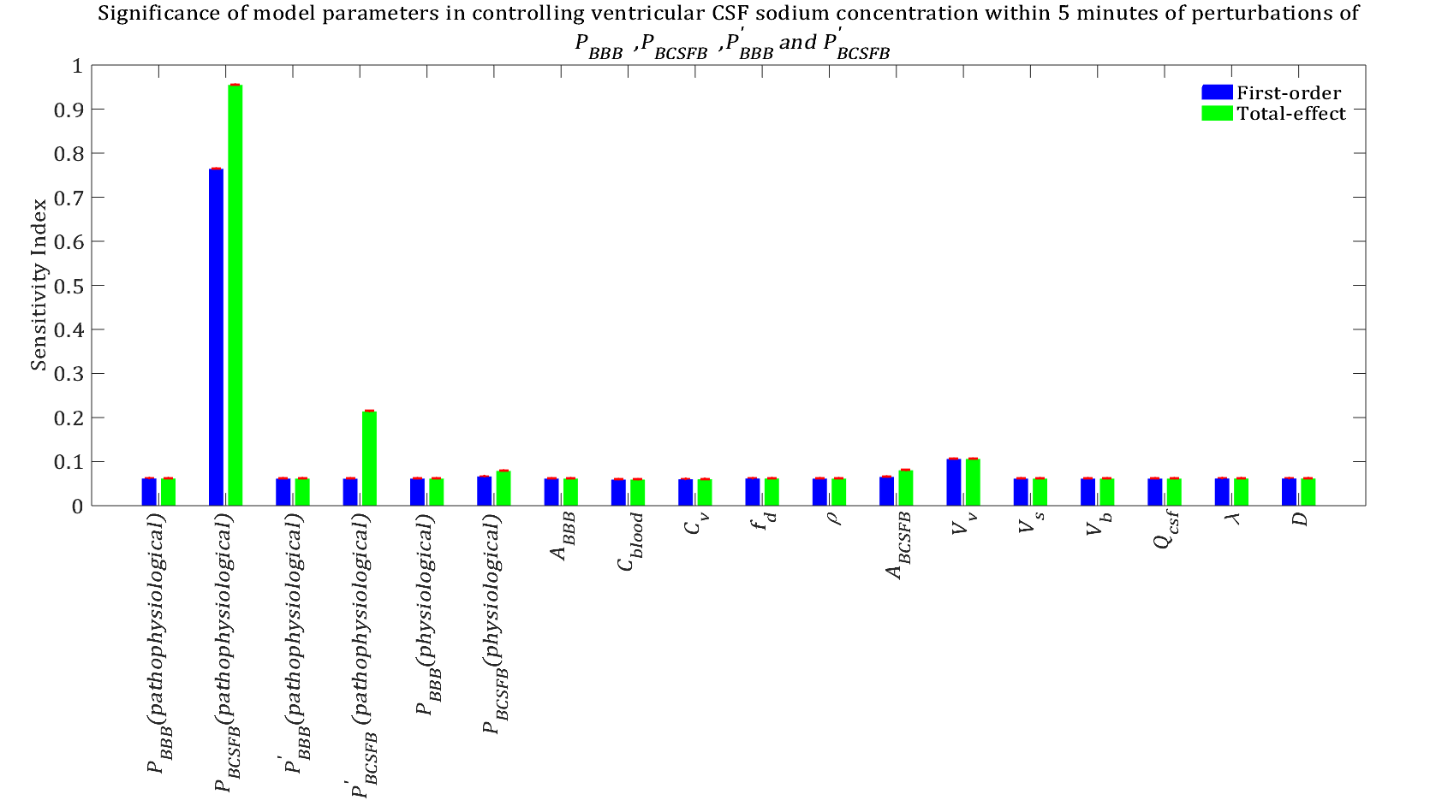


Figure S5. Relative significance of the model parameters in controlling ventricular CSF sodium concentration ($C_{v}$) within 5 minutes of the perturbation onset ($t_{max}=5 min$). The blue bars represent first-order sensitivity indices, while the green bars show the total-effect sensitivity indices. The error bars, shown in red, indicate the bootstrap confidence intervals (95% confidence intervals) of the mean values.


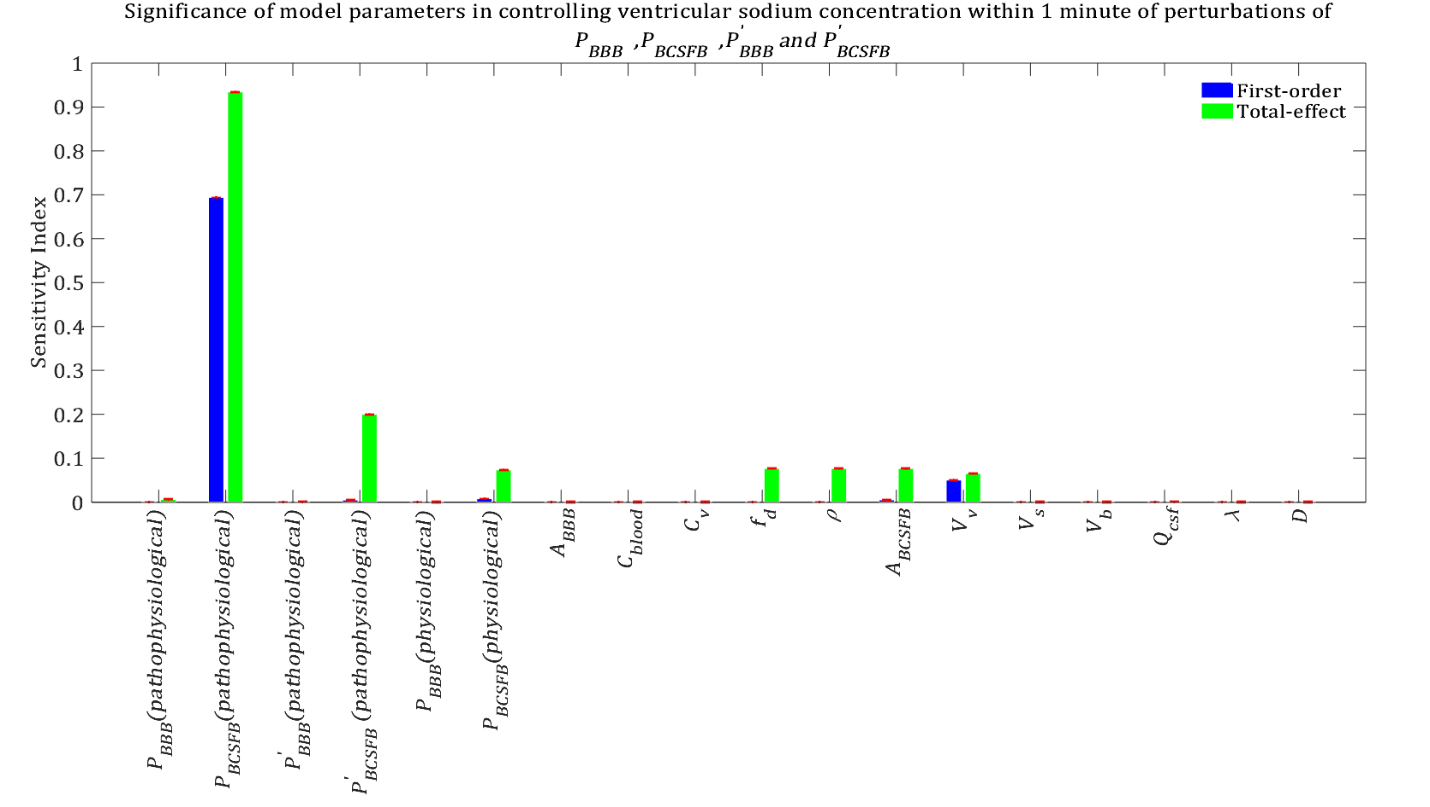


Figure S6. Relative significance of the model parameters in controlling ventricular CSF sodium concentration ($C_{v}$) within 1 minute of the perturbation onset ($t_{max}=1 min$). The blue bars represent first-order sensitivity indices, while the green bars show the total-effect sensitivity indices. The error bars, shown in red, indicate the bootstrap confidence intervals (95% confidence intervals) of the mean values.


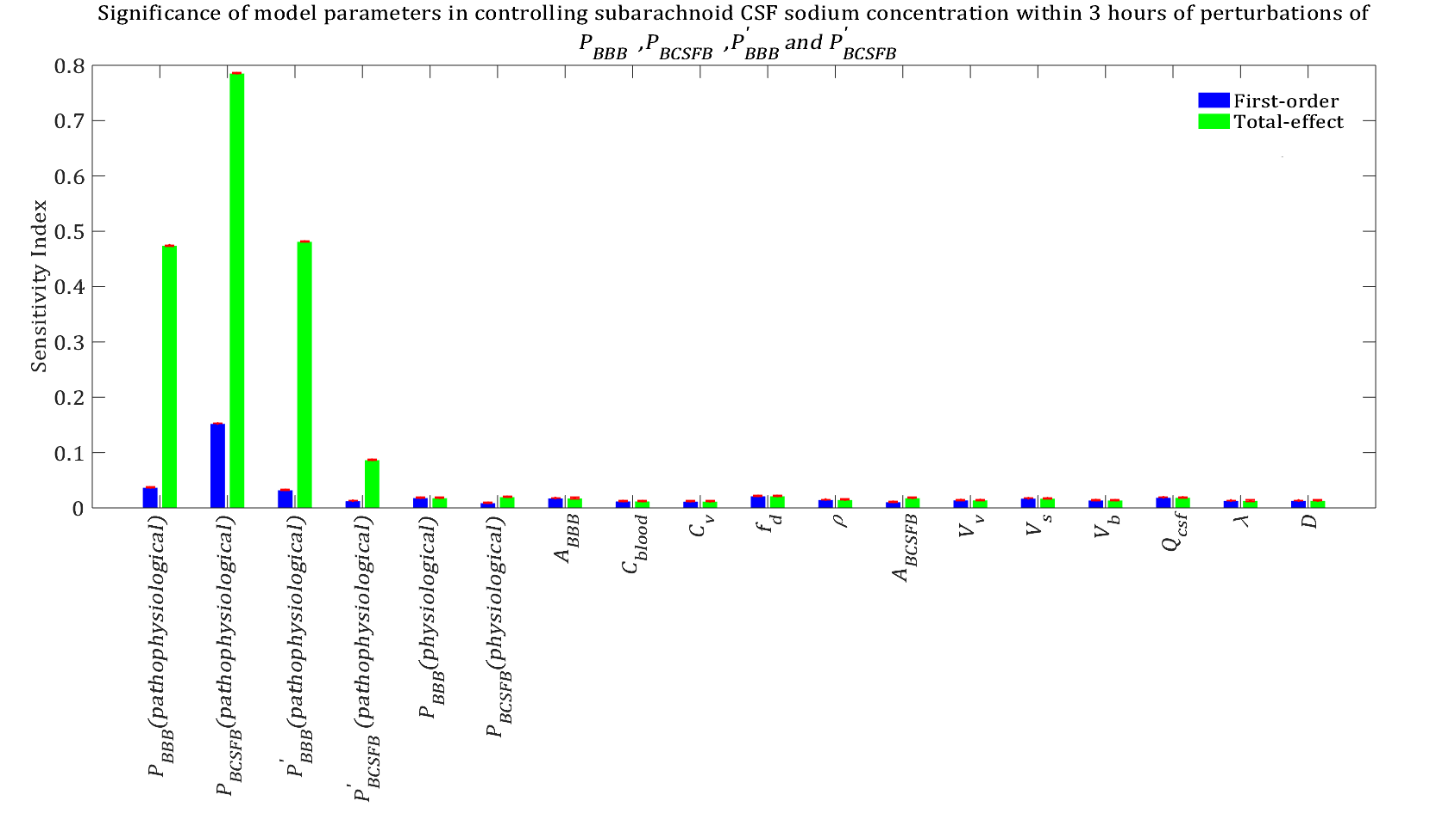


Figure S7. Relative significance of the model parameters in controlling subarachnoid CSF sodium concentration ($C_{s}$) within 3 hours of the perturbation onset ($t_{max}=3 h$). The blue bars represent first-order sensitivity indices, while the green bars show the total-effect sensitivity indices. The error bars, shown in red, indicate the bootstrap confidence intervals (95% confidence intervals) of the mean values.


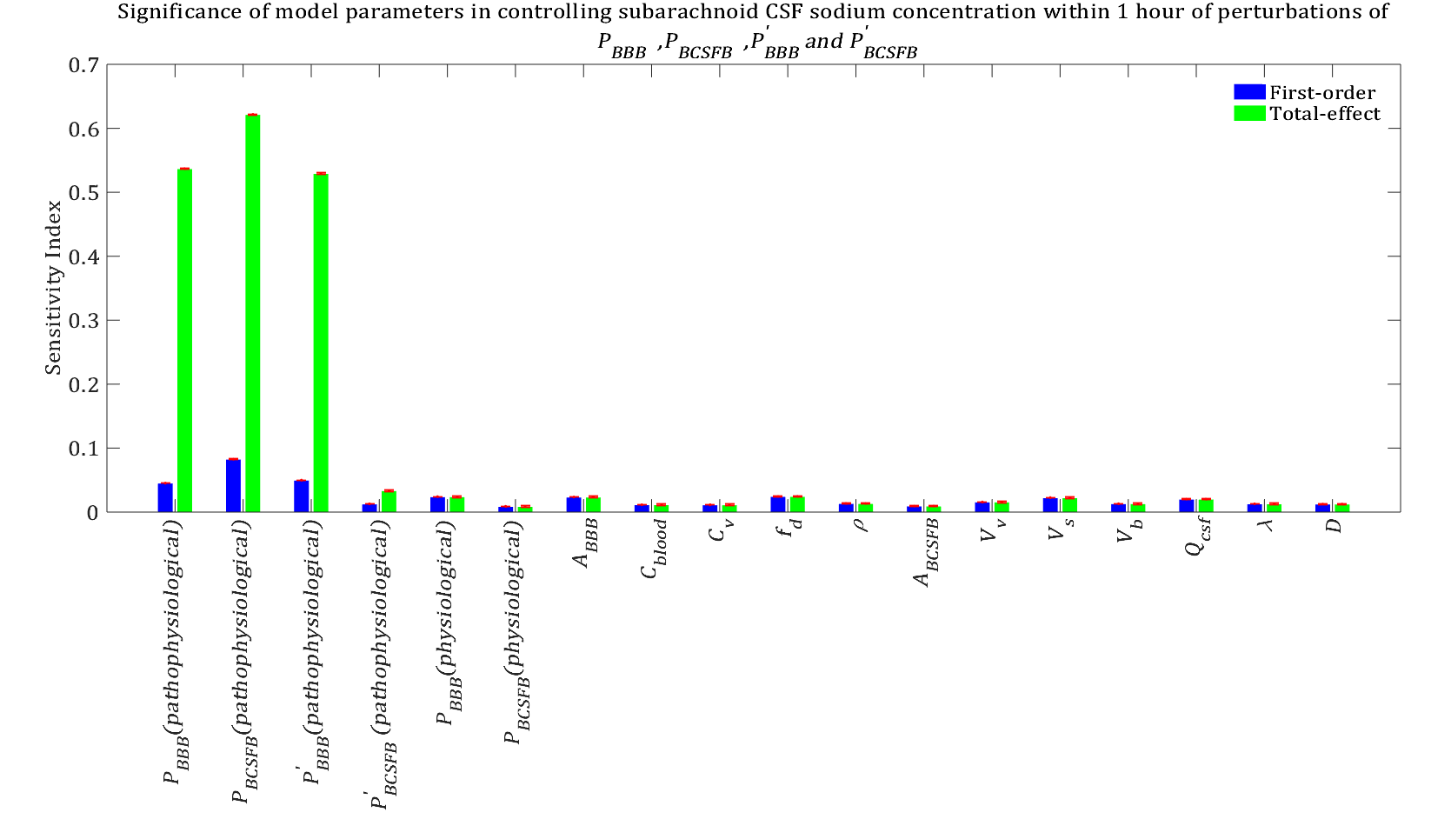


Figure S8. Relative significance of the model parameters in controlling subarachnoid CSF sodium concentration ($C_{s}$) within 1 hour of the perturbation onset ($t_{max}=1 h$). The blue bars represent first-order sensitivity indices, while the green bars show the total-effect sensitivity indices. The error bars, shown in red, indicate the bootstrap confidence intervals (95% confidence intervals) of the mean values.


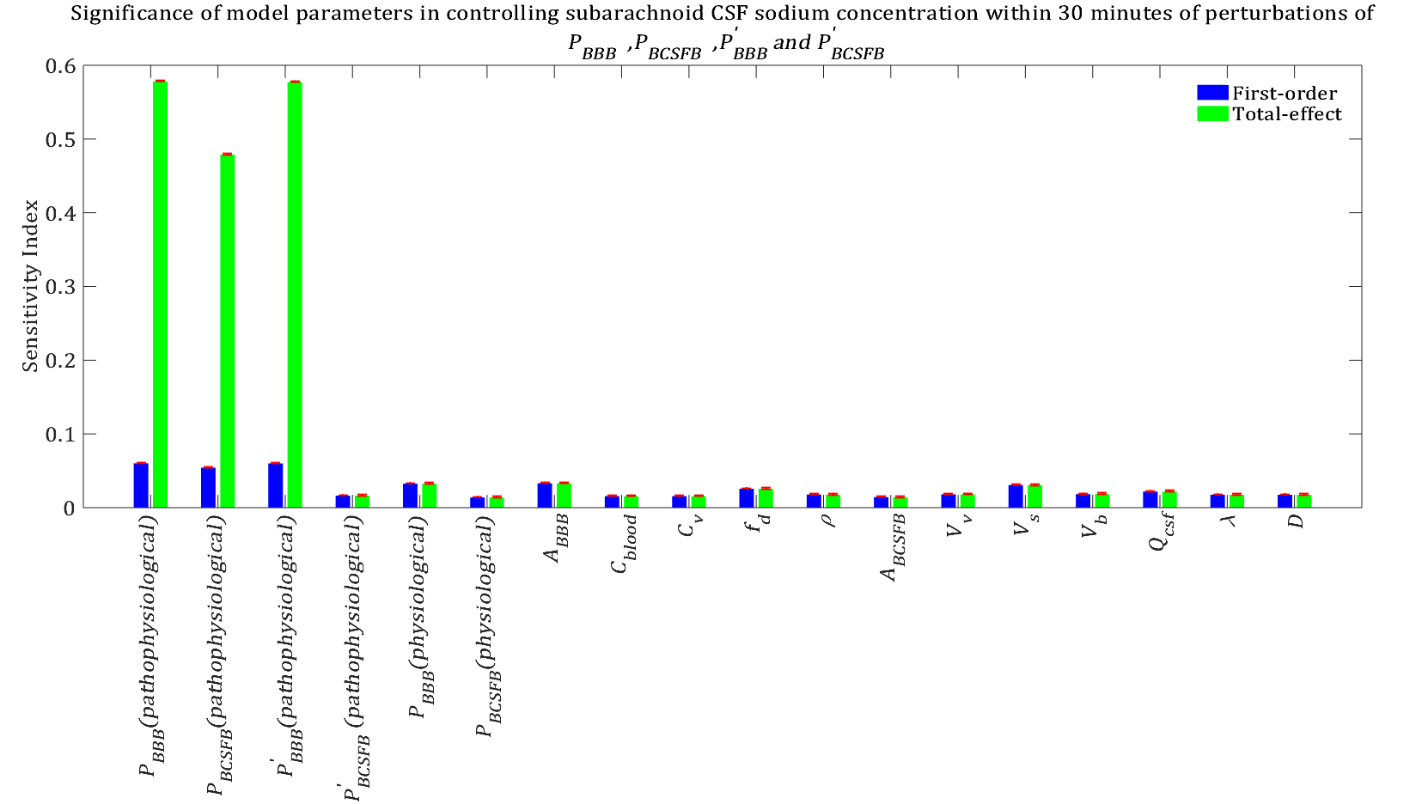


Figure S9. Relative significance of the model parameters in controlling subarachnoid CSF sodium concentration ($C_{s}$) within 30 minutes of the perturbation onset ($t_{max}= 30 min$). The blue bars represent first-order sensitivity indices, while the green bars show the total-effect sensitivity indices. The error bars, shown in red, indicate the bootstrap confidence intervals (95% confidence intervals) of the mean values.


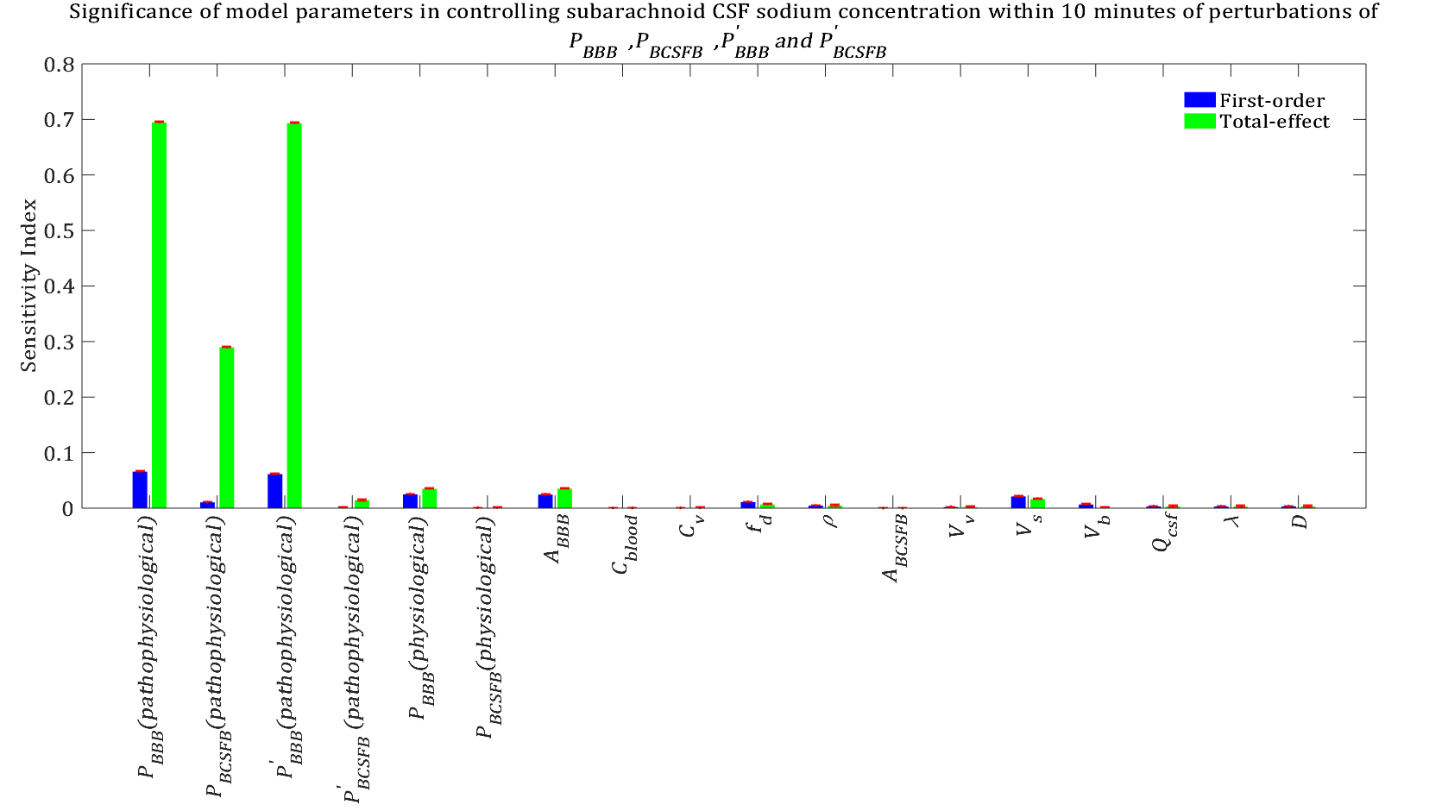


Figure S10. Relative significance of the model parameters in controlling subarachnoid CSF sodium concentration ($C_{s}$) within 10 minutes of the perturbation onset ($t_{max}=10 min$). The blue bars represent first-order sensitivity indices, while the green bars show the total-effect sensitivity indices. The error bars, shown in red, indicate the bootstrap confidence intervals (95% confidence intervals) of the mean values.


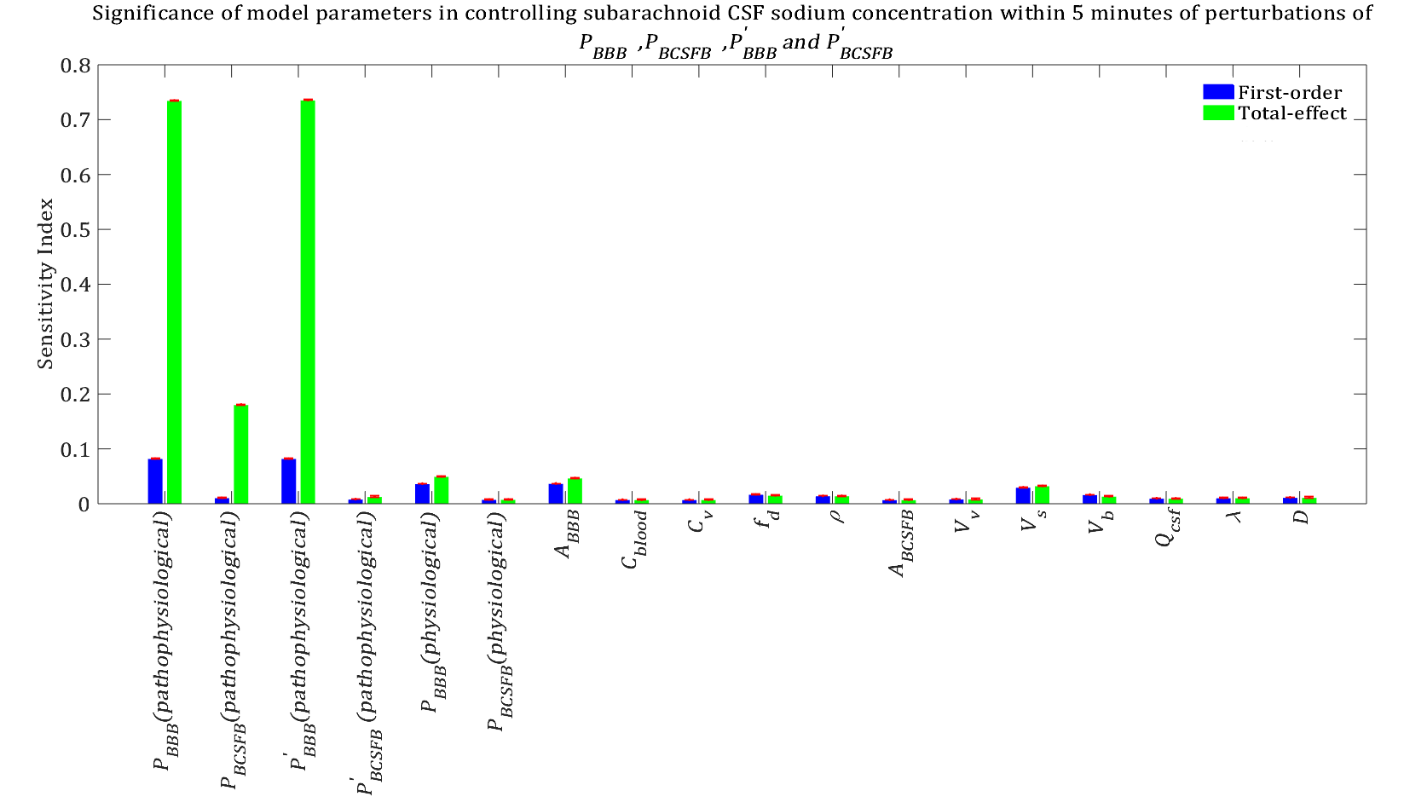


Figure S11. Relative significance of the model parameters in controlling subarachnoid CSF sodium concentration ($C_{s}$) within 5 minutes of the perturbation onset ($t_{max}=5 min$). The blue bars represent first-order sensitivity indices, while the green bars show the total-effect sensitivity indices. The error bars, shown in red, indicate the bootstrap confidence intervals (95% confidence intervals) of the mean values.


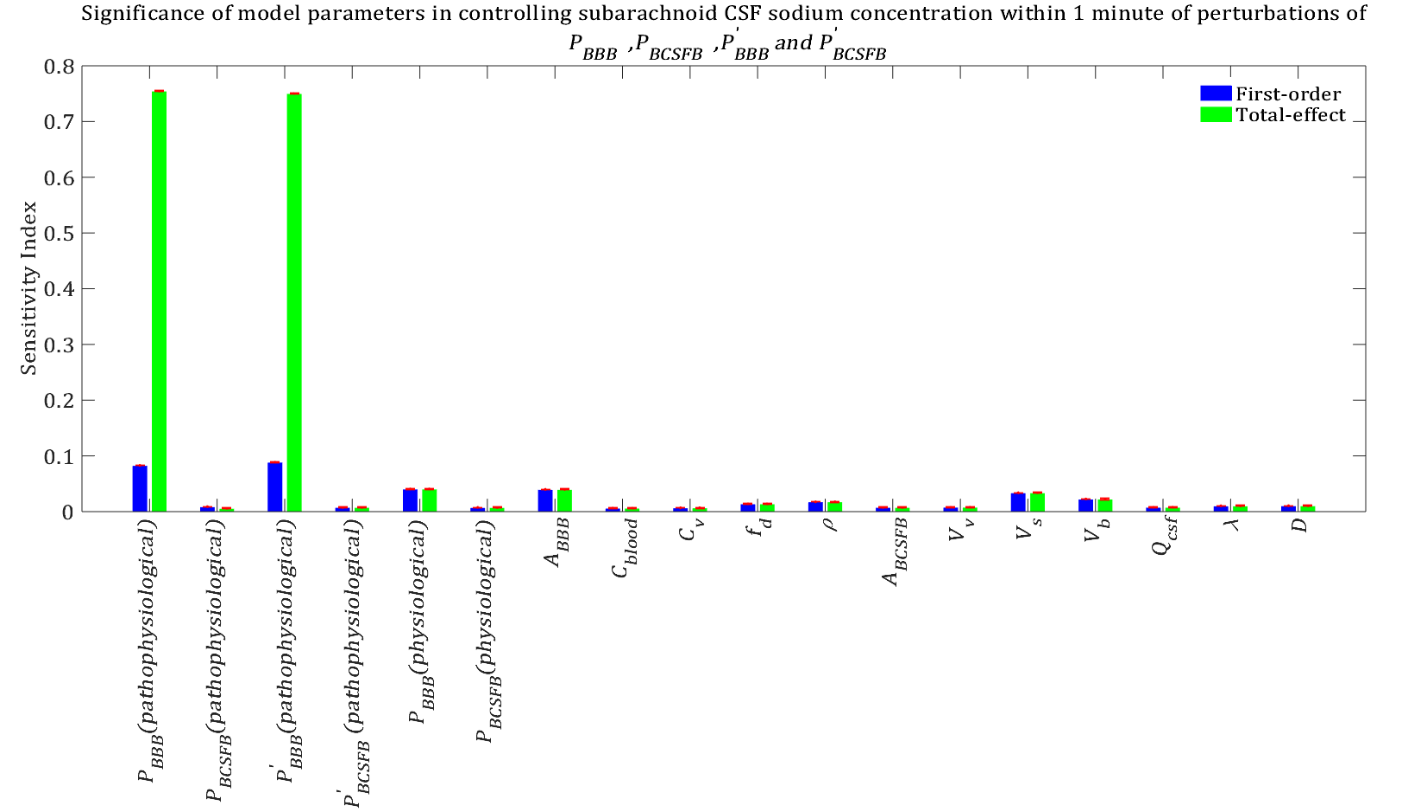


Figure S12. Relative significance of the model parameters in controlling subarachnoid CSF sodium concentration ($C_{s}$) within 1 minute of the perturbation onset ($t_{max}=1 min$). The blue bars represent first-order sensitivity indices, while the green bars show the total-effect sensitivity indices. The error bars, shown in red, indicate the bootstrap confidence intervals (95% confidence intervals) of the mean values.


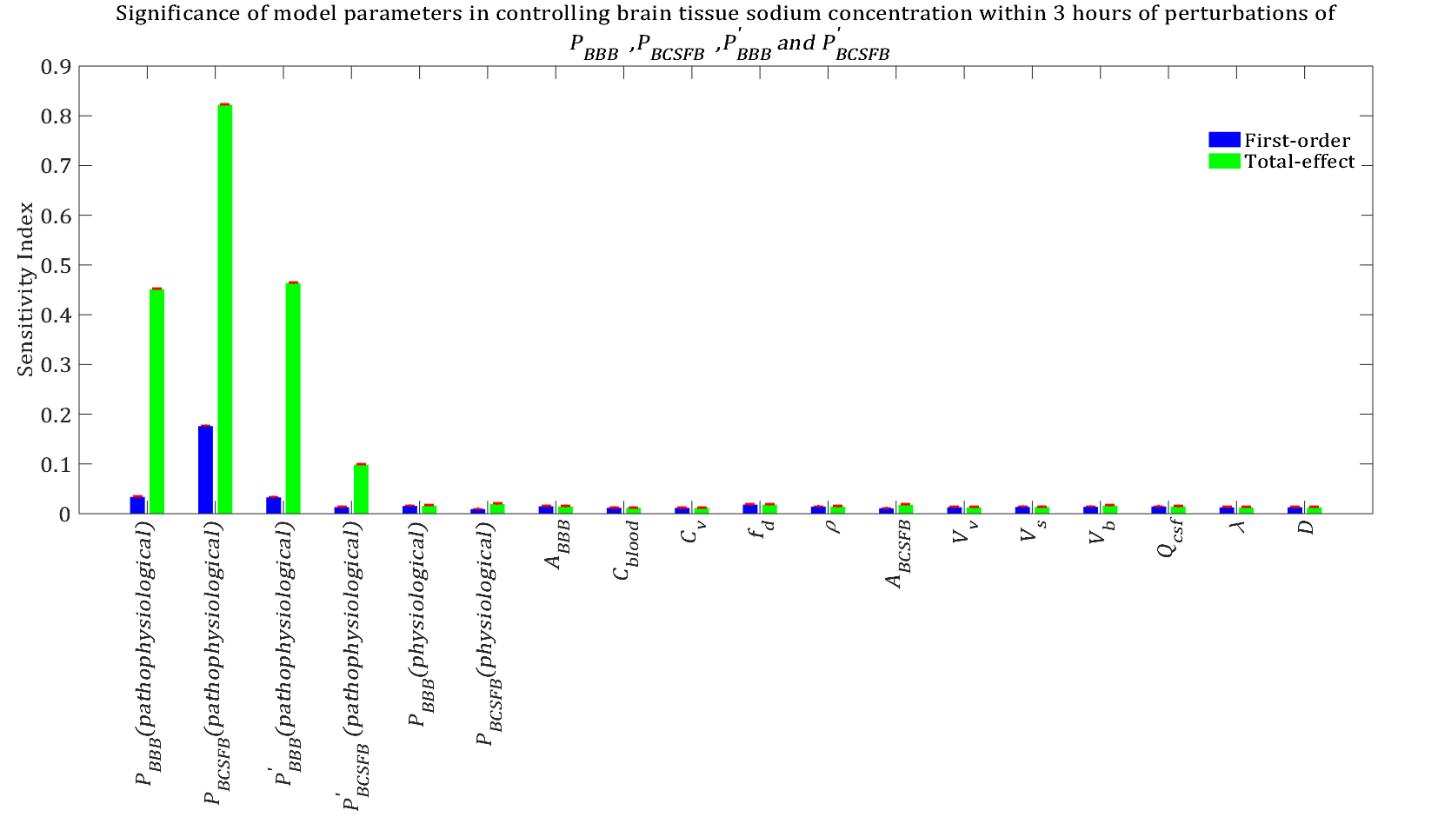


Figure S13. Relative importance of the model parameters in controlling brain tissue sodium levels within 3 hours of the perturbation onset ($t_{max}=3 h$). The blue bars represent first-order sensitivity indices, while the green bars show the total-effect sensitivity indices. The error bars, shown in red, indicate the bootstrap confidence intervals (95% confidence intervals) of the mean values.


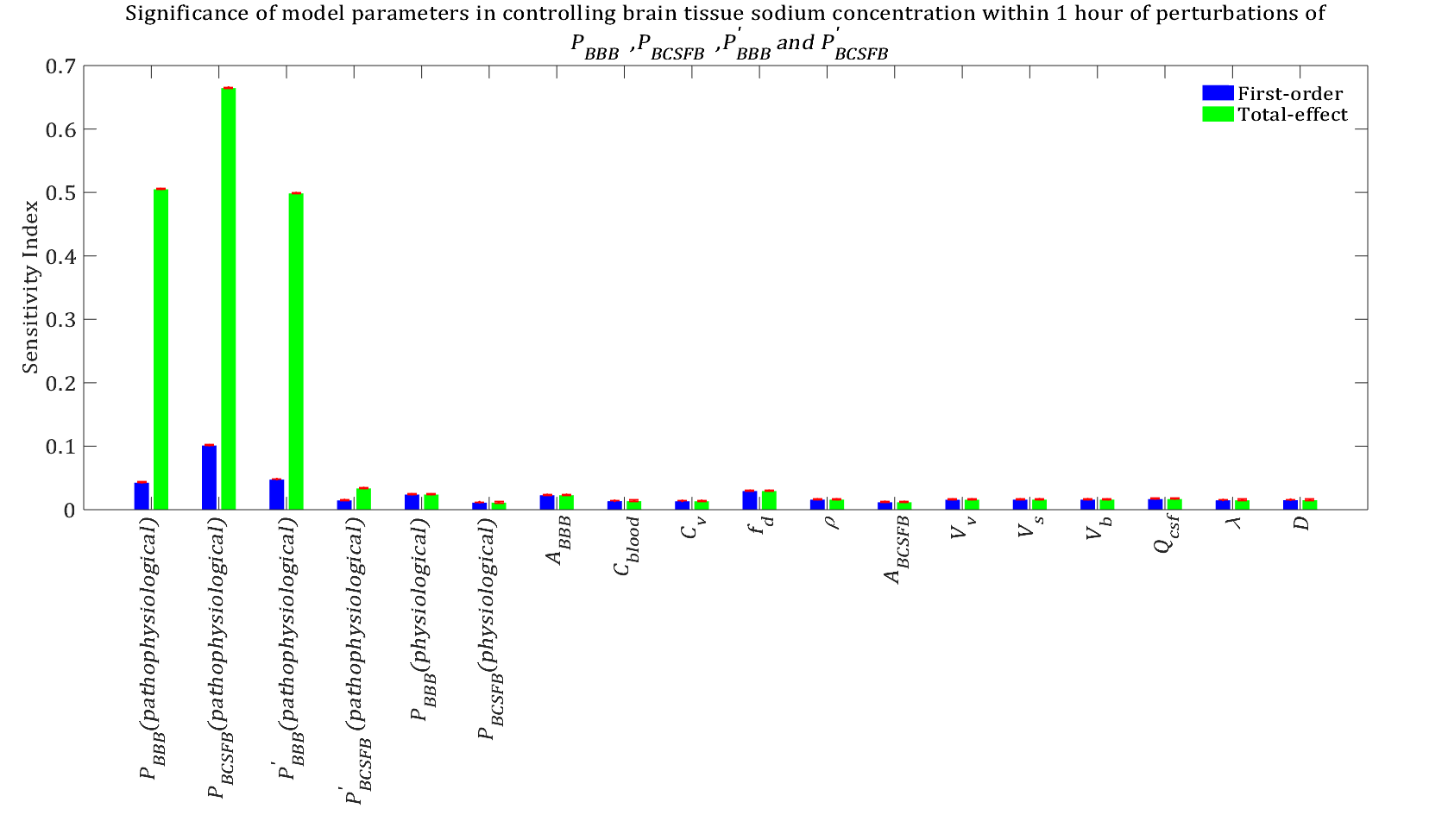


Figure S14. Relative importance of the model parameters in controlling brain tissue sodium levels within 1 hour of the perturbation onset ($t_{max}=1 h$). The blue bars represent first-order sensitivity indices, while the green bars show the total-effect sensitivity indices. The error bars, shown in red, indicate the bootstrap confidence intervals (95% confidence intervals) of the mean values.


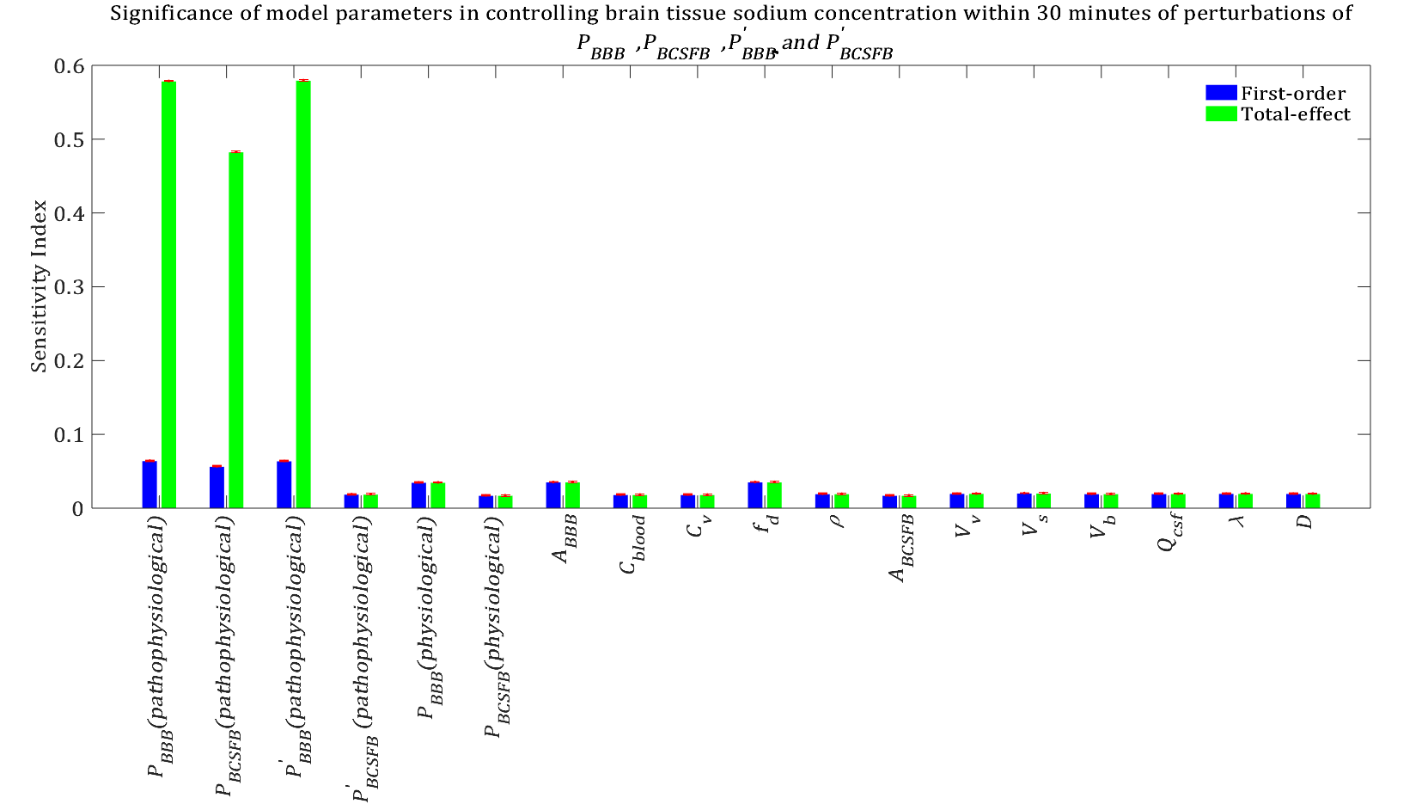


Figure S15. Relative importance of the model parameters in controlling brain tissue sodium levels within 30 minutes of the perturbation onset ($t_{max}=30 min$). The blue bars represent first-order sensitivity indices, while the green bars show the total-effect sensitivity indices. The error bars, shown in red, indicate the bootstrap confidence intervals (95% confidence intervals) of the mean values.


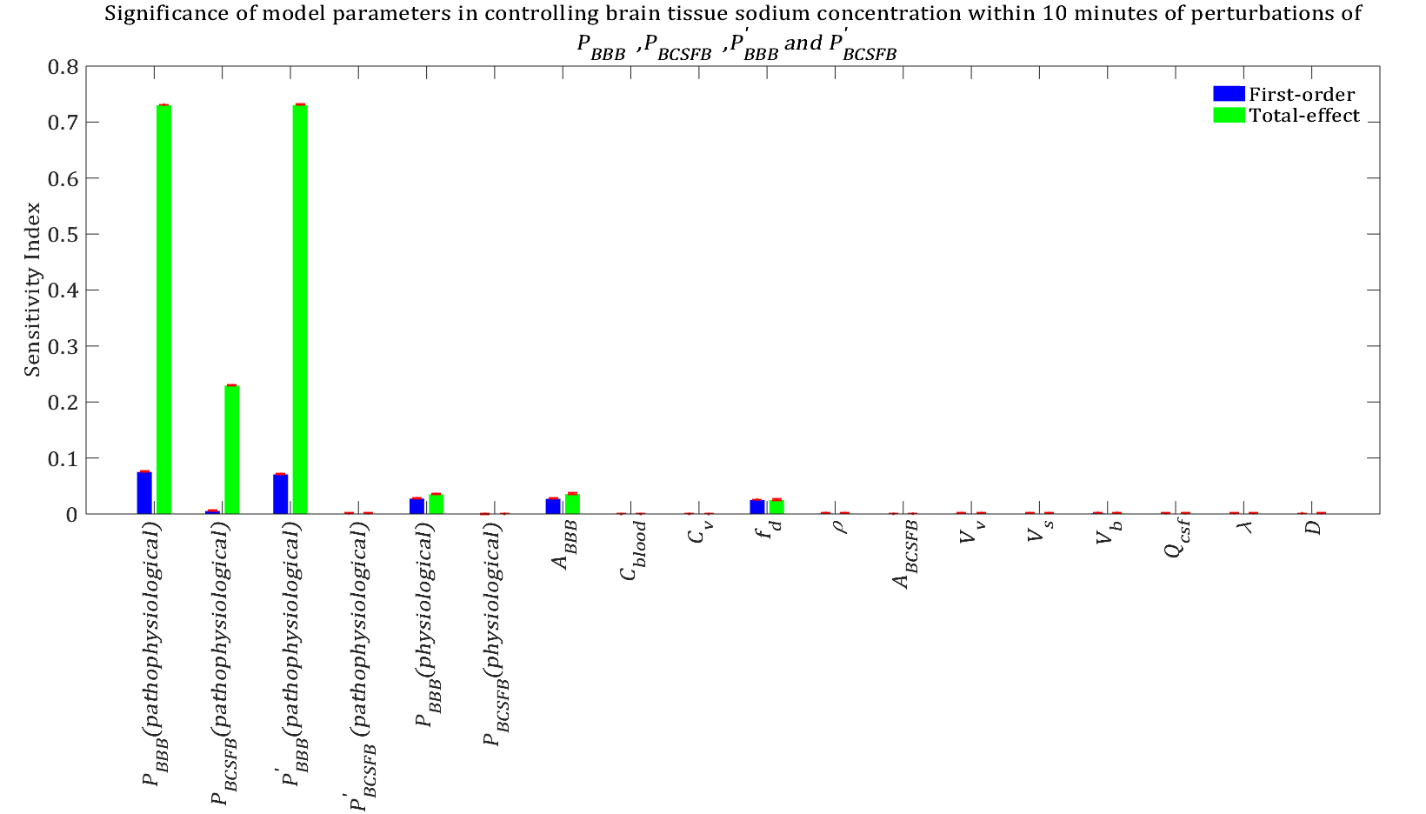


Figure S16. Relative importance of the model parameters in controlling brain tissue sodium levels within 10 minutes of the perturbation onset ($t_{max}=10 min$). The blue bars represent first-order sensitivity indices, while the green bars show the total-effect sensitivity indices. The error bars, shown in red, indicate the bootstrap confidence intervals (95% confidence intervals) of the mean values.


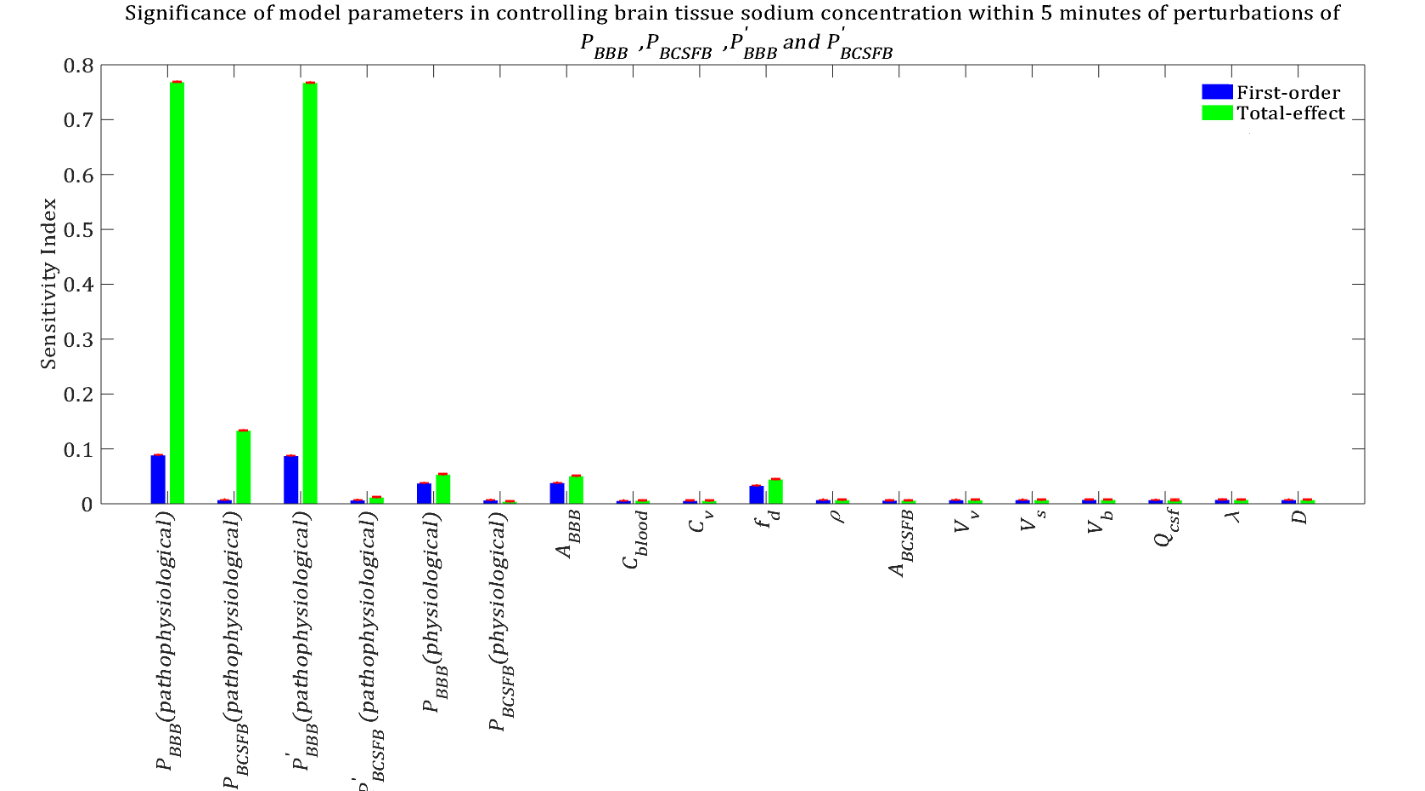


Figure S17. Relative importance of the model parameters in controlling brain tissue sodium levels within 5 minutes of the perturbation onset ($t_{max}=5 min$). The blue bars represent first-order sensitivity indices, while the green bars show the total-effect sensitivity indices. The error bars, shown in red, indicate the bootstrap confidence intervals (95% confidence intervals) of the mean values.


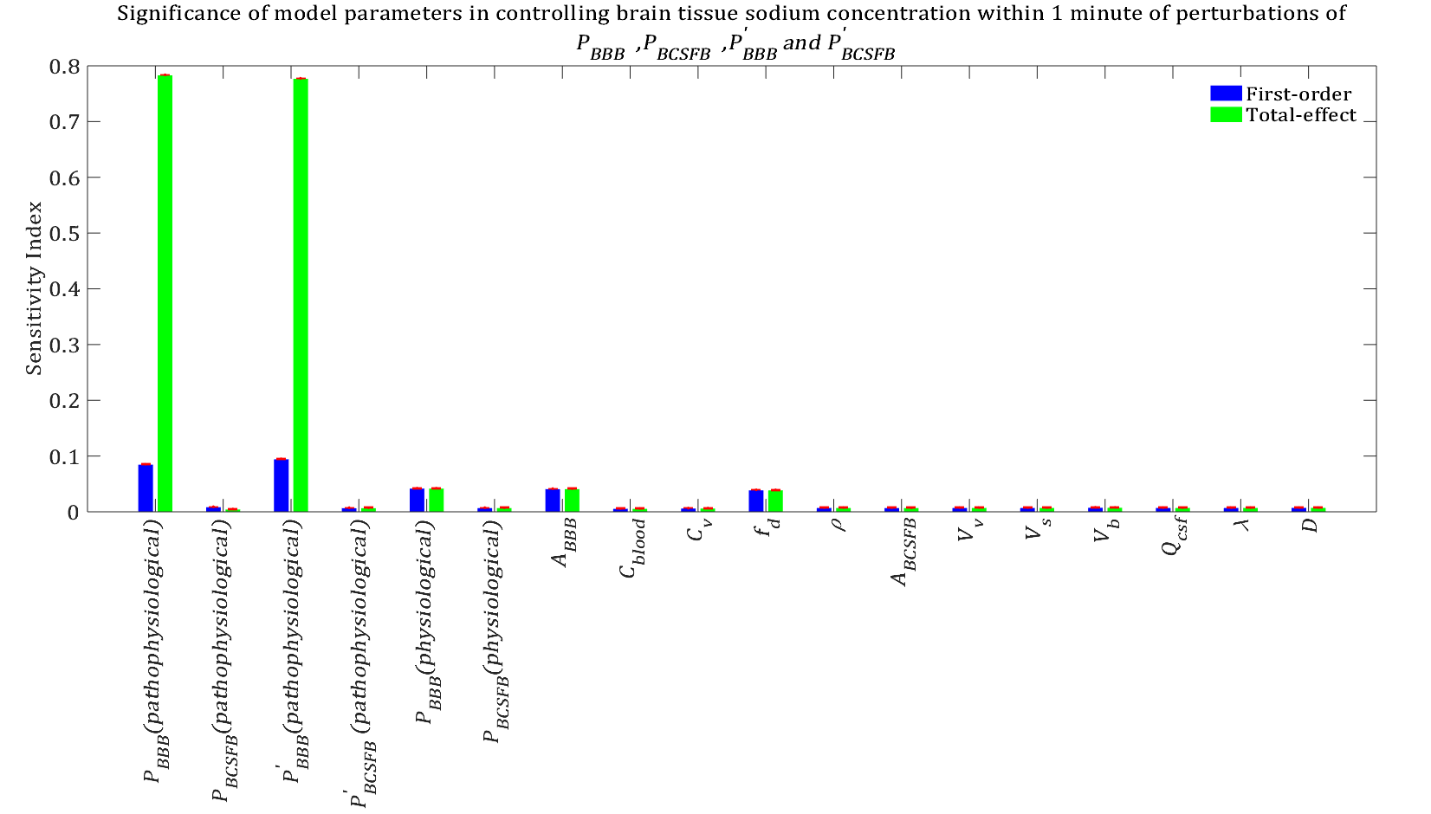


Figure S18. Relative importance of the model parameters in controlling brain tissue sodium levels within 1 minute of the perturbation onset ($t_{max}=1 min$). The blue bars represent first-order sensitivity indices, while the green bars show the total-effect sensitivity indices. The error bars, shown in red, indicate the bootstrap confidence intervals (95% confidence intervals) of the mean values.

**References**

1. Pianosi F, Sarrazin F, Wagener T: **A Matlab toolbox for global sensitivity analysis**. *Environmental Modelling & Software* 2015, **70**:80-5.

2. Tang T, Reed P, Wagener T, Van Werkhoven K: **Comparing sensitivity analysis methods to advance lumped watershed model identification and evaluation**. *Hydrology and Earth System Sciences Discussions* 2006, **3**(6):3333-95.

3. Sin G, Gernaey KV, Neumann MB, van Loosdrecht MC, Gujer W: **Global sensitivity analysis in wastewater treatment plant model applications: prioritizing sources of uncertainty**. *Water research* 2011, **45**(2):639-51.

4. Cosenza A, Mannina G, Vanrolleghem PA, Neumann MB: **Global sensitivity analysis in wastewater applications: A comprehensive comparison of different methods**. *Environmental modelling & software* 2013, **49**:40-52.
